# Multivariate Analysis of Essential Oil Composition of *Artemisia annua* L. Collected from Different Locations in Korea

**DOI:** 10.3390/molecules28031131

**Published:** 2023-01-23

**Authors:** Minji Hong, Minju Kim, Haejung Jang, Sela Bo, Ponnuvel Deepa, Kandhasamy Sowndhararajan, Songmun Kim

**Affiliations:** 1 School of Natural Resources and Environmental Science, Kangwon National University, Chuncheon, Gangwon-do 24341, Republic of Korea; 2 Department of Botany, Kongunadu Arts and Science College, Coimbatore 641029, Tamil Nadu, India

**Keywords:** *Artemisia annua*, chemotype, essential oil, Gae-ddong-ssuk, multivariate analysis

## Abstract

*Artemisia annua* L. is distributed throughout the world and it is an important medicinal plant in Korea to treat various human diseases. Recently, *A. annua* has also been considered to be an effective ethnobotanical drug against COVID-19. *A. annua* contains an appreciable amount of essential oil with different biological properties. However, the composition of essential oils in aromatic plants can be varied depending on several factors, including geographic, genetic, ecological, etc. Hence, the present study aimed to investigate the chemical diversity of essential oils of Korean *A. annua* collected from different locations in Korea by multivariate analysis. For this purpose, the seeds of *A. annua* were collected from 112 different locations in Korea and were grown under the same environmental conditions. Except for nine individuals which decayed during the cultivation, essential oils were isolated from the aerial parts of 103 *A. annua* individuals (AEOs) using the steam distillation extraction method, and their chemical compositions were determined by GC-MS analysis. Furthermore, a multivariate analysis was performed to distinguish the difference between 103 individuals of *A. annua* based on their essential oil compositions. The yield of *A. annua* essential oils ranged from 0.04 to 1.09% (*v*/*w*). Based on the GC-MS data, *A. annua* individuals were grouped into six chemotypes such as artemisia ketone, camphor, β-cubebene, eucalyptol, α-pinene, and β-selinene. The multivariate analysis results revealed that Korean *A. annua* could be largely grouped into three clusters such as artemisia ketone, eucalyptol, and β-selinene. Among 35 components selected for principal component analysis (PCA), PC1, PC2, and PC3 accounted for 82.55%, 8.74%, and 3.62%, respectively. Although all individuals of *A. annua* were cultivated under the same environmental conditions, there is an intraspecific chemical diversity that exists within Korean native species.

## 1. Introduction

The genus *Artemisia* belongs to the family of Asteraceae (Compositae) and it comprises about 500 species [1]. Among them, *Artemisia annua* L. (sweet wormwood or sweet sagewort) is wildly distributed in Asian countries, mainly in China, Japan, and Korea, and it is now naturalized in North American and European countries [2,3,4]. The Korean name of *A. annua* is Gae-ddong-ssuk, which means smell like dog’s excretion when the leaves were rubbed [5]. In Korea, *A. annua* has been used in traditional systems of medicine for hundreds of years [6]. *A. annua* is widely known for its effective anti-malarial component, artemisinin (a sesquiterpene lactone) [7]. Recently, *A. annua* has been in the limelight as an effective ethnobotanical drug against COVID-19 [8]. Previous studies showed that 100 types of chemical components were identified from *A. annua* [9].

In the past few decades, many studies reported that *A. annua* essential oil (AEO) has a variety of biological properties, including antimicrobial, anticancer, anti-inflammatory, antinociceptive, anti-obesity, antioxidant, antipyretic, etc. Further studies reported on the utilization of AEO in aromatherapy, cosmetics, fragrances, groceries, and pharmaceutics [3,7,10,11,12]. The essential oils of *A. annua* are mainly comprised of monoterpenoids and sesquiterpenes. However, the profile of essential oils exhibited great variations in the three major components, artemisia ketone, 1,8-cineole, and camphor, depending on the geographical origin of the plant [2,13,14]. Zhang et al. [9] also reported variations in the main secondary metabolites of *A. annua* such as arteannuin B, artemisinin, artemisinic acid, and scopoletin, according to different geographical locations in China.

Plants within the same species exhibit some morphological differences. However, the variability in the chemical composition of essential oils has been reported, depending on various factors such as geographical origins, cultivation conditions, stage of maturity, harvesting season, genotype, etc. [2,12,13]. AEOs collected from different geographical regions showed markedly different compositions. For instance, artemisia ketone was the major component in Chinese, French, and Indian AEOs. In the case of Iranian AEO, camphor (48.0%) was the most abundant component but showed variations in its concentration (2.8–64%). The essential oils isolated from North American *A. annua* that exhibited artemisia ketone (35.7–68.0%) and eucalyptol (22.8–31.5%) were major components with different proportions [2]. In Tajikistan, *A. annua* samples collected from three different locations exhibited camphor (32.5–58.9%) camphene (4.5–8.4%), eucalyptol (13.7–17.8%), and α-pinene (1.9–7.3%) as major components [3]. Therefore, it is important to understand the chemical composition of essential oils of *A. annua* collected from different geographical regions. Multivariate analysis is an important statistical analysis method to determine and classify the chemical or morphological characteristics of plant species by cluster analysis and principal component analysis [15,16,17]. Previously, Radulović et al. [18] grouped AEOs into four classes based on multivariate analysis. In another study, essential oils from *A. annua* of different origins cultivated in Finland were divided into four classes according to cluster analysis [19]. Hence, the multivariate analysis offers insight into the distribution of essential oil components in plant populations.

It is well understood that the chemical components of AEOs vary significantly based on geographical location. There is no study about variations in the chemical composition of essential oils within Korean populations of *A. annua*. Hence, the present study aimed to demonstrate the variation in the chemical composition of essential oils from Korean *A. annua* individuals. To do this, we collected seeds of *A. annua* from different sites in Korea and cultivated the plants under the same environmental conditions, and analyzed AEOs, using gas chromatography and mass spectrometry analysis (GC-MS). Finally, we performed a multivariate analysis based on the chemical profile of AEOs to classify chemotypes and identify the chemical diversity of *A. annua* individuals in Korea. 

## 2. Results and Discussion

### 2.1. Yield and Color of Korean A. annua Essential Oils

**Figure 1:**
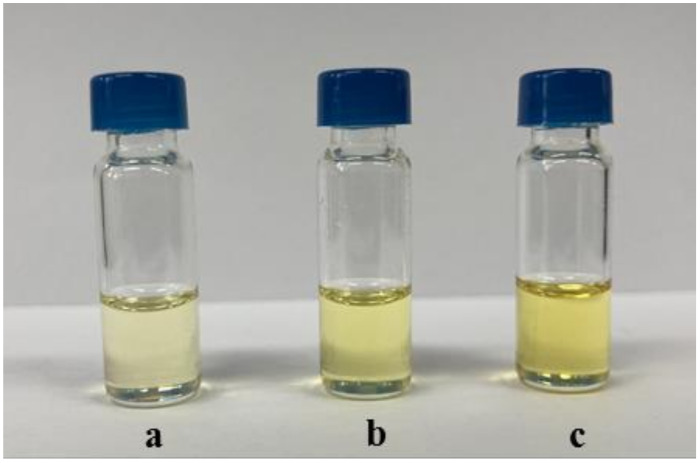
The color classification of *A. annua* essential oils. (**a**) pale yellow; (**b**) yellow; (**c**) dark yellow.

*A. annua* is one of the most useful aromatic plants found around the world. Many researchers reported that AEO components, such as mono-/sesqui-terpenoids, and other phenolic-derived aromatic compounds have been used in several fields such as food, fragrance, and cosmetics. Essential oils possess a wide range of biological properties owing to the presence of a variety of specialized metabolites [12,20]. Furthermore, *A. annua* is the only recognized source of an effective anti-malarial compound, artemisinin [21]. In this study, 112 *A. annua* seeds were collected from diverse sites in Korea and cultivated in the field under the same environmental conditions. Of these, nine seedlings decayed during the cultivation. The yield and the color of essential oils from the aerial parts of *A. annua* were diverse according to the sampling sites, and the yield (*v*/*w*) ranged from 0.04 to 1.09%. The color of AEOs was classified into pale yellow, yellow, and dark yellow (Figure 1), but was most commonly pale yellow in color. Table 1 shows the extraction yield and color of essential oils from *A. annua* individuals. 

Previous studies reported that the yield of essential oils from *A. annua* significantly varied according to the geographical origin of the plants and their plant parts used for the extraction. Holm et al. [19] reported that the extraction yield from the leaves of AEOs collected in four different countries such as China, Hungary, Italy, and Yugoslavia ranged between 0.4 and 0.9%. In Jwarharti, the yield of AEOs from leaves, petals, and stems collected during the flowering season were 1.5%, 1.8%, and 0.2%, respectively [22]. In Russia, the recovery rate and color of AEOs were also different according to the extraction parts. The yield was 0.7% from the aerial parts and 2.0% from the leaves and inflorescence parts. The color of the oils was yellow to green-yellow [17]. The yield of AEO from the aerial parts collected in Serbia was 0.16% [23]. In Korea, Shin [24] reported that the average yield of essential oil from the dried aerial parts of wild *A. annua* was 0.11%. Bhakuni et al. [25] and Bilia et al. [2] demonstrated that the yield of AEOs was generally between 0.3 and 0.4% (*v*/*w*), but could be as high as 4.0% depending on harvesting time, genotypes, and geographic conditions. 

### 2.2. Chemical Variations of Korean A. annua Essential Oils

**Figure 2:**
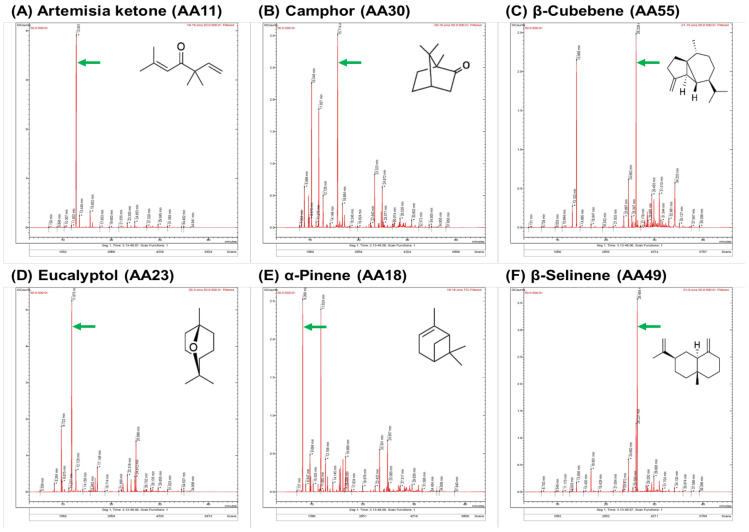
The GC-MS chromatograms of six representative chemotypes of Korean *A. annua* individuals. The peak of major components was marked with a green arrow: (**A**) artemisia ketone; (**B**) camphor; (**C**) β-cubebene; (**D**) eucalyptol; (**E**) α-pinene; (**F**) β-selinene. The sample names of respective chromatograms: AA11, Hongcheon; AA30, Pyeongtaek; AA55, Gwangju; AA23, Chuncheon; AA18, Danyang; AA49, Sejong.

The GC-MS data demonstrated that 103 individuals of Korean *A. annua* were classified into 6 chemotypes according to the predominant components in each essential oil (Table 2). A total of 178 chemical constituents were identified in 103 individuals of AEOs based on the RI value and mass spectral data. Among them, the most dominant chemotype was artemisia ketone (75 individuals), followed by β-selinene (17 individuals), β-cubebene (five individuals), eucalyptol (four individuals), camphor (one individual), and α-pinene (one individual) chemotypes. Furthermore, Figure 2 shows chromatograms of representative major components chosen for six chemotypes of *A. annua*. It was observed that the content of monoterpenoids was higher than the sesquiterpenoids in most of the AEOs. The essential oils of Korean *A. annua* individuals markedly differed both qualitatively and quantitatively.

Similar to the present study, previous studies revealed that artemisia ketone, camphor, caryophyllene, eucalyptol, and α-pinene are the major compounds of AEOs [2,13,26]. Hwang et al. [14] identified 34 compounds in Korean AEO, and the major compounds were eucalyptol (20.6%), germacrene D (19.3%), and caryophyllene (11.4%). On the other hand, Shin [24] reported that among the 85 chemicals contained in Korean AEO, caryophyllene oxide (11.7%), caryophyllene (7.5%), camphor (7.3%), 1,8-cineole (5.0%), and borneol (4.0%) were principal components. Bilia et al. [2] grouped AEOs (aerial parts) in accordance with the major compound and its content as Chinese (artemisia ketone, 64.0%), French (artemisia ketone, 2.8–55.0%; eucalyptol, 1.2–11.6%; germacrene D, 15.0%), Indian (artemisia ketone, 11.5–58.8%), Iranian (camphor, 48.0%; eucalyptol, 9.4%), North American (artemisia ketone, 35.7–68.0%; eucalyptol, 22.8–31.5%), and Vietnamese (camphor, 3.3–21.8%; germacrene D, 0.3–18.9%). In a recent study, Liu et al. [27] found that the most abundant components of AEO collected in China were artemisia ketone (70.6%), α-caryophyllene (5.1%), and germacrene D (3.8%).

However, Tzenkova et al. [28] reported that Bulgarian AEO obtained from the aerial parts mainly contained sesquiterpenoids (67.4%), followed by monoterpenoids (18.0%), and the most abundant component was α-caryophyllene (24.7%). A recent study reported that the major constituents of AEOs collected from three different locations in Tajikistan were camphor (32.5–58.9%), camphene (4.5–8.4%), eucalyptol (13.7–17.8%), and α-pinene (1.9–7.3%) [3]. Another study found that the essential oils obtained from five *Artemisia* species including *A. annua* were dominated by either monoterpenes or sesquiterpenes according to the species and their geographical origin. The most abundant components identified in the essential oils of five *Artemisia* species were β-pinene, chamazulene, germacrene D, camphor, pinocarvone, and thuja-2,4(10)-diene [23]. 

In the case of AEO from the root part, *cis*-arteannuic alcohol (25.9%) was the major component [22]. The most abundant component in the Romanian AEO was camphor (17.74%), followed by α-pinene, germacrene D, 1,8-cineole, trans-β-caryophyllene, and artemisia ketone [29]. In the case of AEO from Tuscany, the major components were camphor (25.2%), 1,8-cineole (20%), and artemisia ketone (12.5%) [30]. It was reported that the flowering top of AEO contained a higher amount of camphor (22.6%), followed by artemisia ketone (17.3%) and 1,8-cineole (15.8%) [31]. These studies clearly indicated the variations in the AEOs according to their geographical origin. In contrast to many other studies, this study could identify the chemotypes of sesquiterpenoids, either β-cubebene or β-selinene, from Korean *A. annua*. It is well known that the essential oil composition can change from plant to plant within the same species. In general, various abiotic and biotic factors in addition to postharvest treatments affect the plant’s secondary metabolite production [32]. 

The major component, especially artemisia ketone, is an irregular monoterpene with green herbaceous fragrance, used in the perfume and cosmetic industries [33]. Camphor has also been extensively used as a fragrance and food flavorant. It is used for the treatment of minor muscle pains and as a skin penetration enhancer. Camphor possesses several biological properties such as insecticidal, antimicrobial, anti-nociceptive, and anticancer activities [34,35]. Another major component, eucalyptol (1,8-cineole), showed anti-inflammatory and antioxidant properties. In addition, eucalyptol is used for the treatment of respiratory and cardiovascular diseases [36]. α-Pinene is one of the most important monoterpenes used in the fragrance and flavor industry and has been used for the treatment of respiratory tract infections for several decades. α-Pinene has antibacterial, insecticidal, antioxidant, and anti-cancer properties [37]. β-cubebene and β-Selinene are also important sesquiterpene hydrocarbons in AEOs.

### 2.3. Multivariate Analysis

Multivariate analysis is one of the extensively used techniques to describe possible relationships between essential oils and their chemical compositions [38]. Out of 178 essential oil components, 35 components that appeared in over 50 individuals of *A. annua* were selected for multivariate analysis (Supplementary Table S1). Artemisia ketone, artemisia alcohol, camphor, caryophyllene, caryophyllene oxide, and α-pinene were reported as the major components of AEOs [3,12,19]. In this study, these components were also included in 35 common chemicals.

#### 2.3.1. Cluster Analysis

**Figure 3:**
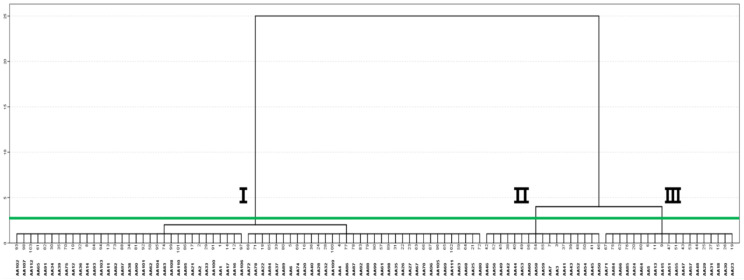
Dendrogram shows three groups of 103 *A. annua* individuals based on their essential oil components.

Figure 3 indicates a dendrogram of Korean *A. annua* individuals based on their essential oil components. The result of cluster analysis demonstrated that Korean *A. annua* individuals could be classified into three major groups. In the group I, *A. annua* individuals which have the highest content of artemisia ketone with a ratio of monoterpenoids content of over 64% were included. Individuals of *A. annua* with a higher amount of sesquiterpenoids such as β-selinene were placed under Group II. Group III consisted of *A. annua* individuals with a similar proportion of mono-/sesqui-terpenoid contents. In Table 3, the chemical characteristics of individuals of Korean *A. annua* were summarized according to different groups.

Previous studies also compared and analyzed *A. annua* individuals based on the composition of major essential oil components. Radulović et al. [18] reported that AEOs could be categorized into four classes (Class 1: camphor and camphor/eucalyptol; Class 2: artemisia ketone/eucalyptol/α-pinene and artemisia ketone/camphor/eucalyptol; Class 3: artemisia ketone/camphor/germacrene D; and Class 4: β-caryophyllene/germacrene D and artemisia ketone/β-caryophyllene/eucalyptol/germacrene D). Sharopov et al. [3] also suggested that AEOs were classified into three types, such as group–I camphor/eucalyptol, group II–camphor, and group III–artemisia ketone. Based on a PCA of essential oils, five *Artemisia* species including *A. arborescens*, *A. campestris*, *A. lobelii*, *A. annua*, and *A. absinthium* were separated into camphor, chamazulene and α-pinene [23]. Holm et al. [19] divided seven batches of *A. annua*, which were native to different countries, into four clusters according to their essential oil compositions, and it was found that the genotype was strongly correlated with the chemical compositions. Charles et al. [26] also reported that the great diversity in AEOs’ constituents was based upon genetic differences, and suggested that the determination of the essential oil composition is important for improving its quality.

#### 2.3.2. Principal Component Analysis (PCA)

**Figure 4:**
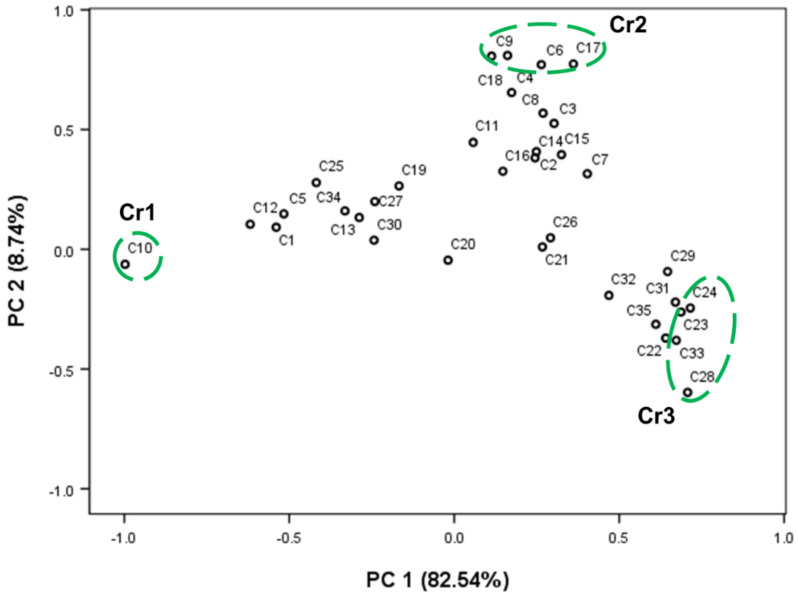
The loading plot of PCA shows the correlations between 35 common chemicals and principal components. Chemicals in green circle: Cr1 had a high correlation with PC1 (negative: artemisia ketone (C10); Cr2 had a high correlation with PC2 (positive: α-terpinene (C6); eucalyptol (C9); terpinen-4-ol (C17); α-terpineol (C18); Cr3 had a high correlation with PC1 (positive: β-cubebene (C23); β-caryophyllene (C24); β-selinene (C28); α-muurolol (C33)).

PCA eases the raw data’s complexity, but retains most of the information to highlight the variation [39]. As a result of PCA, 35 principal components (PCs) were sorted into three PCs. PC1, PC2, and PC3 occupied 82.55%, 8.74%, and 3.62% of the proportion of variance (Table 4), respectively. The cumulative proportion (%) of PC1 and PC2 accounted for 91.28%. Thus, PC1 and PC2 can be determined as the main principal components, and these were described intensively in this section. Table 4 shows the correlation coefficient between 35 common chemicals of *A. annua* and each principal component. PC1 is positively correlated with the contents of β-caryophyllene (C24; 0.716), β-selinene (C28; 0.708), β-cubebene (C23; 0.687), and α-muurolol (C33; 0.673), whereas it showed a high negative correlation with the contents of artemisia ketone (C10; −0.998) and artemisia alcohol (C12; −0.619). PC2 showed a high positive correlation with the contents of α-terpineol (C18; 0.810), eucalyptol (C9; 0.807), terpinene-4-ol (C17; 0.774), and α-terpinene (C6; 0.771). The chemicals which have a high correlation with PC1 and PC2 were summarized in Table 5. Moreover, Figure 4 shows a loading plot for the correlation of 35 common chemicals in AEOs with PC1 (*x*-axis) and PC2 (*y*-axis).

**Figure 5:**
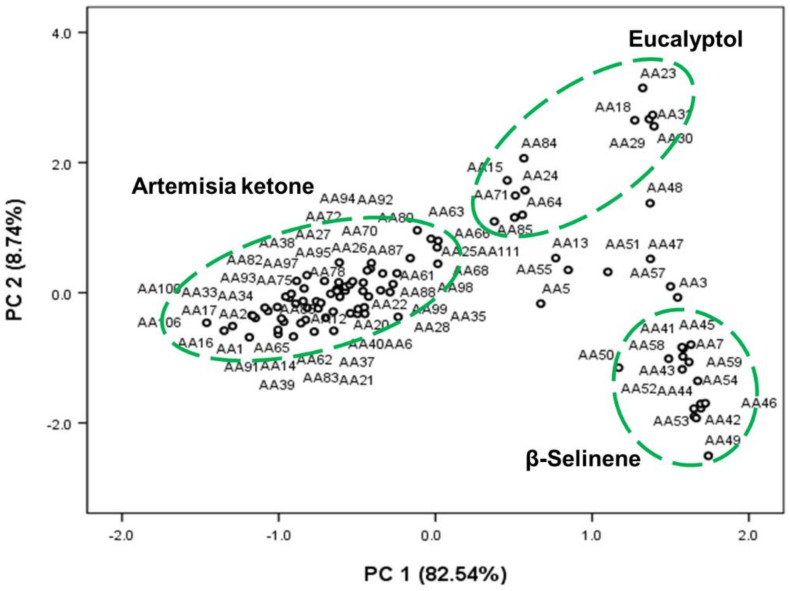
The scatter plot of PCA shows *A. annua* individuals’ relativeness based on 35 common chemicals: green circles indicate three major groups of the *A. annua* individuals: artemisia ketone, eucalyptol, and β-selinene groups.

Using the PCA scores (PC1 and PC2), a scatter plot of Korean native *A. annua* individuals was constructed as shown in Figure 5. Based on 35 principal components, all individuals of *A. annua* could be largely classified into three groups, artemisia ketone, eucalyptol, and β-selinene. *A. annua* individuals, which have a high positive correlation with PC1 (high contents of β-selinene, β-caryophyllene, and β-cubebene or low content of artemisia ketone) were composed of AA7, AA41, AA42, AA43, AA44, AA45, AA46, AA49, AA50, AA52, AA53, AA54, AA56, AA58, AA59, and AA60. A total of 12 *A. annua* individuals such as AA15, AA18, AA23, AA24, AA29, AA30, AA31, AA64, AA66, AA71, AA84, and AA85 were correlated with PC2 (high contents of eucalyptol and α-terpineol). Zhigzhitzhapova et al. [17] reported that AEOs could be divided conditionally into two groups (Asian and European) based on PCA using their chemical composition data available in the literature.

#### 2.3.3. Correlation Analysis

A correlation coefficient table showed the correlation between 35 common chemicals in Korean AEOs individuals (Supplementary Table S2). The results of correlation analysis indicated that all chemicals exhibited a complicate correlation between them. Therefore, chemicals that showed statistical significance at the 1% level and had a correlation value over 0.7 were explained in this section.

α-Pinene showed a high correlation with pinocarveol (0.890 **) and pinocarvone (0.797 **), camphene with β-pinene (0.831 **) and camphor (0.936 **), and β-pinene with limonene (0.706 **) and camphor (0.749 **). Yomogi alcohol had a high positive correlation with artemisia alcohol (0.802 **), α-terpinene with terpinene-4-ol (0.916 **) and α-terpineol (0.729 **), and α-terpineol with eucalyptol (0.874 **) and terpinene-4-ol (0.746 **). In addition, l-pinocarveol was highly correlated with pinocarvone (0.879 **), β-cubebene with β-caryophyllene (0.788 **) and γ-elemene (0.800 **), and α-muurolol with benzyl isovalerate (0.713 **) and lanceol (0.860 **). However, there is a strong negative correlation between artemisia ketone and β-caryophyllene (−0.703 **). Other chemicals showed a low correlation with each other, and these results would contribute to understanding the relationship between Korean *A. annua* individuals and their common chemicals (Supplementary Table S2). 

In the multivariate analysis, the result of cluster analysis revealed the classification of 103 Korean *A. annua* individuals into three major groups based on the ratio of monoterpene and sesquiterpene compounds. In PCA, the selected 35 components were sorted into three PCs and the cumulative proportion of PC1 and PC2 accounted for 91.28%. Furthermore, Korean populations of *A. annua* were broadly classified into three groups such as artemisia ketone, eucalyptol, and β-selinene according to the PCA scatter plot.

The data of this study indicated that there were significant differences in the chemical components and their ratios of essential oils of 103 *A. annua* individuals collected from different regions in Korea. Attention should be paid to the variations in the chemical compositions within species, and differences in their biological properties need to be further investigated.

## 3. Materials and Methods

### 3.1. Collection and Cultivation of Korean A. annua Seeds

**Figure 6:**
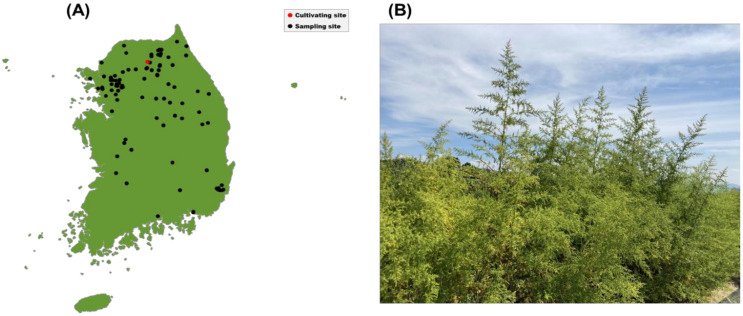
(**A**) Sampling sites of *A. annua* seeds marked as black dots and their cultivation site also marked as a red dot in Korea; (**B**) View of cultivation fields for *A. annua*.

In this study, the seeds of 112 individuals of *A. annua* were collected from different locations in Korea during 2019–2021 and the collection was done with the support of Dr. Jang (Ph. D. of Botany in KNU) (Table 6). The collected seeds were stored at 4 °C and were sown in black seedling trays (128 holes, 17 cm^3^, Seoul-Bio, Korea) filled with horticultural media in April 2022. Every seedling tray was kept for 35–36 days in a glassed greenhouse at the Gangwon-do Agricultural Research and Extension Services (GARES) with constant temperature (23–25 °C) and humidity (50%). In early May, all seedlings of *A. annua* which reached a 3.5 leaf base were planted at the cultivation fields located in Chuncheon, Gangwon-do, Korea (N 37°55′45.4″; E 127°43′44.2″) (Figure 6). Except for the nine dead individuals that decayed during cultivation, 103 *A. annua* individuals were grown until the flowering stage and were harvested for the extraction of essential oils.

### 3.2. Extraction of Essential Oils

The harvested samples were stored in the cold room at 4 °C prior to the extraction of essential oils. In total, 103 AEOs were extracted from fresh aerial parts of *A. annua* individuals by the steam distillation extraction method. For each *A. annua* individual, one kilogram of the fresh sample was extracted at 100 °C for 90 min by the steam distillation apparatus (EssenLab Plus, Hanil Lab Tech Co, Ltd., Yangju, Korea). The yield of AEOs (%, *v*/*w*) was calculated as the volume (mL) of each essential oil per 1 kg plant sample. After the extraction, AEOs were purified using anhydrous sodium sulfate (Na_2_SO_4_) and were kept in the refrigerator at 4 °C.

### 3.3. Identification of Essential Oil Components by GC-MS Analysis

The GC-MS analysis was performed to detect the volatile components in AEOs. A GC-MS instrument (GC: Varian CP-3800 and MS: Varian 1200L, Varian, Palo Alto, CA, USA) was equipped with a fused silica VF-5MS low polarity column (30 m × 0.25 mm × 0.25 μm film thickness; Agilent, Santa Clara, CA, USA). The carrier gas used was helium at the flow rate of 1 mL/min. The GC conditions were as follows: the inlet temperature was 250 °C; the oven temperature was programmed for 50–250 °C, an increasing rate of 5 °C/min with an initial hold time of 5 min and a final hold time of one minute; the injection volume was 1 μL with split ratio 10:1. The MS conditions were as follows: the ionization mode was electron ionization; electron beam energy was set to 70 eV; the ion source temperature was 200 °C; and the mass scan range was set to 50–500 m/z. The identification of chemicals in AEOs was compared with the mass spectra data of NIST library version 3.0 and their retention indices (RI) relative to a homologous series of n-alkanes (C_8_–C_20_) with those reported in the literature data [40].

### 3.4. Statistical Analysis

For GC-MS analysis, the essential oil components from *A. annua* individuals were subjected to hierarchical cluster analysis and principal component analysis (PCA). For this purpose, the GC-MS data of 103 samples of *A. annua* essential oil were integrated into one data point (raw data). The chemical components in the raw data were arranged in ascending order according to their retention RI value. Only components in a concentration above 1.0% were considered for further statistical analysis. Of these, chemical components detected in over 50 individuals of *A. annua* were selected for PCA analysis. Multivariate and correlation analyses were undertaken based on the common chemical content of AEOs. The cluster analysis and dendrogram were constructed based on the results of PCA [15]. All statistical analyses were carried out by IBM SPSS version 26 (IBM Corp., Chicago, IL, USA).

## 4. Conclusions

The results demonstrate that the essential oils obtained from 103 individuals of Korean *A. annua* showed significant chemical diversity. Based on the chemical compositions and their relative abundances, 103 *A. annua* essential oils could be classified into six chemotypes such as artemisia ketone, camphor, β-cubebene, eucalyptol, α-pinene, and β-selinene. Furthermore, a multivariate analysis based on GC-MS data allowed us to identify variability among the populations of Korean *A. annua.* The cluster analysis and PCA revealed that *A. annua* individuals were divided into three large groups: artemisia ketone, eucalyptol, and β-selinene. These major components may be used as biomarkers to determine the origin of *A. annua* populations. These results explain that the intraspecific variations in the essential oil compositions of Korean native *A. annua* may be due to the influence of genetic diversity. Hence, further genetic analysis studies are warranted to confirm the observed variations within *A. annua* populations. 

## Figures and Tables

**Table 1:** The yield (*v*/*w* %) and color of essential oils from Korean *A. annua* individuals.

No.	Samples	Yield (%)	Color	No.	Samples	Yield (%)	Color	No.	Samples	Yield (%)	Color
1	AA1	0.237	Y	36	AA40	0.288	DY	71	AA78	0.689	DY
2	AA2	0.527	DY	37	AA41	0.046	Y	72	AA80	0.645	PY
3	AA3	0.142	DY	38	AA42	0.107	DY	73	AA82	0.697	PY
4	AA4	0.311	Y	39	AA43	0.076	DY	74	AA83	0.664	Y
5	AA5	0.723	PY	40	AA44	0.038	PY	75	AA84	0.647	PY
6	AA6	0.227	DY	41	AA45	0.038	Y	76	AA85	0.617	Y
7	AA7	0.088	DY	42	AA46	0.041	DY	77	AA86	0.500	DY
8	AA11	0.360	Y	43	AA47	0.060	Y	78	AA87	0.745	Y
9	AA12	0.377	DY	44	AA48	0.058	DY	79	AA88	0.690	PY
10	AA13	0.223	DY	45	AA49	0.059	Y	80	AA89	0.561	PY
11	AA14	0.440	PY	46	AA50	0.039	DY	81	AA90	0.932	PY
12	AA15	0.335	Y	47	AA51	0.064	Y	82	AA91	1.088	PY
13	AA16	0.330	PY	48	AA52	0.037	DY	83	AA92	0.629	PY
14	AA17	0.435	Y	49	AA53	0.049	Y	84	AA93	0.792	PY
15	AA18	0.273	DY	50	AA54	0.063	DY	85	AA94	0.895	PY
16	AA20	0.194	DY	51	AA55	0.085	DY	86	AA95	0.983	PY
17	AA21	0.367	DY	52	AA56	0.041	PY	87	AA96	1.063	PY
18	AA22	0.257	DY	53	AA57	0.059	PY	88	AA97	1.060	PY
19	AA23	0.426	Y	54	AA58	0.050	PY	89	AA98	0.818	PY
20	AA24	0.490	DY	55	AA59	0.061	Y	90	AA99	0.751	PY
21	AA25	0.533	Y	56	AA60	0.046	PY	91	AA100	0.744	PY
22	AA26	0.492	DY	57	AA61	0.630	Y	92	AA101	0.767	PY
23	AA27	0.305	DY	58	AA62	0.720	PY	93	AA102	0.913	PY
24	AA28	0.526	DY	59	AA63	0.772	PY	94	AA103	0.798	PY
25	AA29	0.519	Y	60	AA64	0.827	PY	95	AA104	0.762	PY
26	AA30	0.213	Y	61	AA65	0.624	PY	96	AA105	0.574	PY
27	AA31	0.369	Y	62	AA66	0.581	PY	97	AA106	0.771	PY
28	AA32	0.137	DY	63	AA67	0.652	PY	98	AA107	0.903	PY
29	AA33	0.380	Y	64	AA68	0.566	Y	99	AA108	0.561	PY
30	AA34	0.355	Y	65	AA69	0.606	PY	100	AA109	0.663	PY
31	AA35	0.311	DY	66	AA70	0.624	PY	101	AA110	0.566	PY
32	AA36	0.267	DY	67	AA71	0.650	PY	102	AA111	0.536	PY
33	AA37	0.214	DY	68	AA72	0.891	PY	103	AA112	0.926	PY
34	AA38	0.361	DY	69	AA74	0.501	Y				
35	AA39	0.337	Y	70	AA75	0.910	PY				

Color–DY: dark yellow; PY: pale yellow; Y: yellow.

**Table 2:** The chemotype classification of Korean *A. annua* individuals based on the major component of essential oils.

Chemotypes	Content Ratio (%)	Samples
Artemisia ketone (75)	20.51–83.82	AA1, AA2, AA4, AA5, AA6, AA11, AA12, AA13, AA14, AA15, AA16, AA17, AA20, AA21 AA22, AA24, AA25, AA26, AA27, AA28, AA32, AA33, AA34, AA35, AA36, AA37, AA38, AA39, AA40, AA61, AA62, AA63, AA64, AA65, AA66, AA67, AA68, AA69, AA70, AA71, AA72, AA74, AA75, AA78, AA80, AA82, AA83, AA85, AA86, AA87, AA88, AA89, AA90, AA91, AA92, AA93, AA94, AA95, AA96, AA97, AA98, AA99, AA100, AA101, AA102, AA103, AA104, AA105, AA106, AA107, AA108, AA109, AA110, AA111, AA112
Camphor (1)	25.05	AA30
β-Cubebene (5)	13.90–22.52	AA47, AA48, AA51, AA55, AA57
Eucalyptol (4)	15.07–43.01	AA23, AA29, AA31, AA84
α-Pinene (1)	21.16	AA18
β-Selinene (17)	20.05–46.29	AA3, AA7, AA41, AA42, AA43, AA44, AA45, AA46, AA49, AA50, AA52, AA53, AA54, AA56, AA58, AA59, AA60

The numbers in the parenthesis denote the total number of samples respective to each chemotype.

**Table 3:** Characteristics of chemical composition for three different clusters of *A. annua* individuals from Korea.

Group	Major Compound	Chemical Characteristics
I	Artemisia ketone	Monoterpenoids content ratio in the essential oil is dominant (monoterpenoids content ratio > 64%; artemisia ketone ratio > 41%).
II	β-Selinene	Sesquiterpenoids content ratio in the essential oil is dominant(sesquiterpenoids content ratio > 37%; β-selinene > 20%)
III	Eucalyptol, β-cubebene	Monoterpenoids and sesquiterpenoids content ratio in the essential oil is similar (monoterpenoids content:sesquiterpenoids content = 1:1)

**Table 4:** Principal component scores of 35 common chemicals in the essential oils of Korean *A. annua* individuals.

No.	Chemical Name	Code	Principal Components		
			PC1	PC2	PC3
1	Santolina triene	C1	−0.540	0.092	−0.066
2	α-Pinene	C2	0.249	0.408	0.088
3	Camphene	C3	0.303	0.526	0.085
4	β-Pinene	C4	0.174	0.654	0.054
5	Yomogi alcohol	C5	−0.516	0.148	0.126
6	α-Terpinene	C6	0.264	**0.771**	−0.115
7	p-Cymene	C7	0.403	0.315	0.186
8	Limonene	C8	0.269	0.569	−0.185
9	Eucalyptol	C9	0.113	**0.807**	−0.523
10	Artemisia ketone	C10	**−0.998**	−0.063	−0.026
11	Sabinene hydrate	C11	0.057	0.446	−0.291
12	Artemisia alcohol	C12	**−0.619**	0.105	0.027
13	3-Isopentenyl isovalerate	C13	−0.288	0.133	−0.094
14	Pinocarveol	C14	0.245	0.382	0.093
15	Camphor	C15	0.325	0.395	0.028
16	Pinocarvone	C16	0.148	0.326	0.089
17	Terpinen-4-ol	C17	0.361	**0.774**	−0.110
18	α-Terpineol	C18	0.161	**0.810**	−0.401
19	3-Hexenyl isovalerate	C19	−0.167	0.265	−0.168
20	α-Longipinene	C20	−0.019	−0.046	−0.293
21	α-Copaene	C21	0.267	0.010	0.229
22	Benzyl isovalerate	C22	0.641	−0.371	−0.006
23	β-Cubebene	C23	**0.687**	−0.262	0.442
24	β-Caryophyllene	C24	**0.716**	−0.245	0.275
25	β-Farnesene	C25	−0.418	0.279	−0.064
26	α-Humulene	C26	0.292	0.048	0.189
27	β-Chamigrene	C27	−0.241	0.199	0.010
28	β-Selinene	C28	**0.708**	−0.598	−0.371
29	γ-Elemene	C29	0.647	−0.093	0.411
30	Butylated hydroxytoluene	C30	−0.243	0.038	0.136
31	δ-Cadinene	C31	0.670	−0.221	0.188
32	Caryophyllene oxide	C32	0.469	−0.193	0.261
33	α-Muurolol	C33	**0.673**	−0.380	0.311
34	Vulgarone B	C34	−0.331	0.160	−0.144
35	Lanceol	C35	0.611	−0.313	0.399
**Proportion of variance (%)**			**82.554**	**8.738**	**3.616**
**Cumulative proportion (%)**			**82.554**	**91.283**	**94.899**

Extraction methods: Principal component analysis; three components were extracted. Bold letters–correlation coefficient was > 0.67 or < −0.61.

**Table 5:** Correlation of common chemicals from *A. annua* essential oils with each principal component (PC1 and PC2).

PC	Correlation	Relevant Chemicals
PC1	Positive (+)	β-Caryophyllene, β-selinene, β-cubebene, α-muurolol
	Negative (−)	Artemisia ketone, artemisia alcohol
PC2	Positive (+)	α-Terpineol, eucalyptol, terpinene-4-ol, α-terpinene

**Table 6:** Collection sites of *A. annua* seeds from different places in Korea.

No.	Sample Code	Sampling Site	No.	Sample Code	Sampling Site	No.	Sample Code	Sampling Site
1	AA1	Wonju	39	AA39	Ulsan	77	AA77	Yanggu
2	AA2	Hwacheon	40	AA40	Bonghwa	78	AA78	Yanggu
3	AA3	Chuncheon	41	AA41	Imsil	79	AA79	Yanggu
4	AA4	Yangyang	42	AA42	Imsil	80	AA80	Yanggu
5	AA5	Sokcho	43	AA43	Jeonju	81	AA81	Yanggu
6	AA6	Goseong	44	AA44	Nonsan	82	AA82	Yanggu
7	AA7	Inje	45	AA45	Daejeon	83	AA83	Seoul
8	AA8	Yanggu	46	AA46	Hanam	84	AA84	Seoul
9	AA9	Yangpyeong	47	AA47	Pyeongtaek	85	AA85	Sungnam
10	AA10	Jeongseon	48	AA48	Sejong	86	AA86	Sungnam
11	AA11	Hongcheon	49	AA49	Sejong	87	AA87	Incheon
12	AA12	Hoengseong	50	AA50	Hanam	88	AA88	Incheon
13	AA13	Pyeongchang	51	AA51	Ulsan	89	AA89	Incheon
14	AA14	Namyangju	52	AA52	Ulsan	90	AA90	Daejeon
15	AA15	Pocheon	53	AA53	Bonghwa	91	AA91	Yeongcheon
16	AA16	Gapyeong	54	AA54	Bonghwa	92	AA92	Mungyeong
17	AA17	Chungju	55	AA55	Gwangju	93	AA93	Bonghwa
18	AA18	Danyang	56	AA56	Yongin	94	AA94	Gimcheon
19	AA19	Jecheon	57	AA57	Hongcheon	95	AA95	Bonghwa
20	AA20	Yeongwol	58	AA58	Seoul	96	AA96	Yeongju
21	AA21	Hongcheon	59	AA59	Seoul	97	AA97	Ulsan
22	AA22	Hongcheon	60	AA60	Samcheok	98	AA98	Ulsan
23	AA23	Chuncheon	61	AA61	Seoul	99	AA99	Ulsan
24	AA24	Chuncheon	62	AA62	Seoul	100	AA100	Ulsan
25	AA25	Hwacheon	63	AA63	Seoul	101	AA101	Changwon
26	AA26	Hwacheon	64	AA64	Seoul	102	AA102	Changwon
27	AA27	Chuncheon	65	AA65	Seoul	103	AA103	Changnyeong
28	AA28	Chuncheon	66	AA66	Seoul	104	AA104	Sacheon
29	AA29	Hongcheon	67	AA67	Seoul	105	AA105	Guri
30	AA30	Pyeongtaek	68	AA68	Anyang	106	AA106	Gapyeong
31	AA31	Yongin	69	AA69	Goyang	107	AA107	Ganghwa
32	AA32	Wonju	70	AA70	Nonsan	108	AA108	Paju
33	AA33	Incheon	71	AA71	Yeoju	109	AA109	Inje
34	AA34	Anyang	72	AA72	Yeoju	110	AA110	Ansan
35	AA35	Seoul	73	AA73	Yanggu	111	AA111	Yongin
36	AA36	Changwon	74	AA74	Yanggu	112	AA112	Chulwon
37	AA37	Ulsan	75	AA75	Yanggu			
38	AA38	Ulsan	76	AA76	Yanggu			

## Data Availability

Not applicable.

## Supplementary Materials

The following supporting information can be downloaded at: https://www.mdpi.com/article/10.3390/molecules28031131/s1, Table S1. The area percent of 35 components in the essential oils of Korean *Artemisia annua* individuals. Table S2. Correlation coefficients between 35 components of essential oils of Korean *Artemisia annua* individuals.

## Sample Availability

Samples of essential oils of *Artemisia annua* are available from the authors.
