# Supplementary material for: Multivariate Analysis of Essential Oil Composition of Artemisia annua L. Collected from Different Locations in Korea
Source: Molecules. 2023 Jan 23;28(3):1131. doi: 10.3390/molecules28031131 (PMC9920137; doi:10.3390/molecules28031131)
Supplement: Supplementary file 1 [file molecules-28-01131-s001.zip › molecules-2166780-supplementary.pdf]

Table S1. The area percent of 35 components in the essential oils of Korean *Artemisia annua* individuals.

Chemical information					*A. annua* individuals																						
RI[a]	RI[b]	Name	Cas no.	Formula	AA1	AA2	AA3	AA4	AA5	AA6	AA7	AA11	AA12	AA13	AA14	AA15	AA16	AA17	AA18	AA20	AA21	AA22	AA23	AA24	AA25	AA26	AA27
908	903	Santolina triene	2153-66-4	$C_{10}H_{16}$	0.64	0.31	0.00	1.32	0.00	0.73	0.00	0.55	0.37	0.32	1.17	1.19	0.21	0.05	0.11	0.17	0.16	1.13	0.00	0.25	0.52	0.20	0.18
939	939	α-Pinene	80-56-8	$C_{10}H_{16}$	0.00	0.38	10.29	0.07	4.75	0.00	0.00	1.90	0.13	14.37	0.00	17.31	0.04	0.00	21.16	0.00	0.09	4.16	1.40	0.83	0.34	0.15	0.20
954	956	Camphene	79-92-5	$C_{10}H_{16}$	0.00	0.00	1.39	0.49	0.06	0.00	0.54	0.62	0.07	0.24	0.00	0.27	0.13	0.00	0.83	0.00	0.22	0.13	0.16	1.66	0.00	0.00	0.00
979	982	β-Pinene	127-91-3	$C_{10}H_{16}$	0.37	0.99	1.58	1.55	0.41	1.46	0.00	2.98	2.31	2.22	4.00	8.41	0.61	0.38	6.53	0.40	0.31	3.17	1.74	4.66	6.72	0.19	0.58
999	997	Yomogi alcohol	26127-98-0	$C_{10}H_{18}O$	0.33	0.72	0.10	0.94	0.54	0.84	0.23	0.60	1.10	0.59	1.63	0.33	0.52	0.54	0.06	0.86	0.47	1.39	0.06	0.70	0.81	0.92	1.06
1017	1020	α-Terpinene	99-86-5	$C_{10}H_{16}$	0.00	0.00	0.15	0.00	0.05	0.14	0.00	0.25	0.00	0.33	0.12	0.08	0.00	0.00	0.83	0.09	0.00	0.00	0.60	0.23	0.19	0.10	0.17
1094	1029	p-Cymene	99-87-6	$C_{10}H_{14}$	0.00	0.00	0.15	0.00	0.13	0.08	0.00	0.08	0.00	0.21	0.30	0.12	0.00	0.00	0.35	0.25	0.00	0.00	0.16	0.00	0.00	0.06	0.07
1029	1034	Limonene	138-86-3	$C_{10}H_{16}$	0.00	0.00	0.00	0.19	0.04	0.04	0.00	0.03	0.00	0.00	0.00	0.38	0.00	0.00	0.34	0.00	0.06	0.11	0.26	0.25	0.00	0.00	0.00
1131	1038	Eucalyptol	470-82-6	$C_{10}H_{18}O$	0.66	2.96	2.72	2.37	1.64	2.66	4.00	4.36	0.99	4.05	0.00	13.43	0.73	1.66	19.62	1.38	1.58	2.40	43.01	18.70	13.67	10.28	11.54
1062	1064	Artemisyl ketone	546-49-6	$C_{10}H_{16}O$	76.19	74.64	0.00	63.27	23.77	56.69	0.00	68.32	65.48	20.51	70.87	27.27	80.43	78.91	3.73	57.24	61.43	53.81	3.65	25.53	41.65	53.09	58.97
1070	1075	Sabinene hydrate	15537-55-0	$C_{10}H_{18}O$	0.00	0.32	0.15	0.07	0.00	0.11	0.85	0.10	0.00	0.00	0.00	0.79	0.10	0.00	2.69	0.19	0.00	0.00	0.31	0.10	0.18	0.05	0.00
1083	1083	Artemisia alcohol	27644-04-8	$C_{10}H_{18}O$	2.22	2.35	0.07	3.18	0.54	2.44	0.38	2.63	2.88	1.16	4.34	1.42	3.84	0.53	0.05	3.25	1.90	3.52	0.05	1.51	2.32	1.07	0.98
1114	1114	3-Isopentenyl isovalerate	54410-94-5	$C_{10}H_{18}O_2$	0.00	0.00	0.00	0.00	0.04	0.00	0.00	0.15	0.00	0.00	0.05	0.06	0.00	0.00	0.07	0.44	0.12	0.00	0.09	0.08	0.00	0.00	0.11
1139	1148	Pinocarveol	547-61-5	$C_{10}H_{16}O$	0.00	0.18	2.68	0.00	1.42	0.00	0.00	0.30	0.00	3.51	0.00	2.42	0.00	0.00	2.62	0.00	0.21	0.44	0.28	0.29	0.00	0.08	0.12
1146	1155	Camphor	76-22-2	$C_{10}H_{16}O$	0.00	0.00	5.99	3.57	0.11	0.64	7.85	0.05	0.56	0.34	0.00	0.16	2.39	0.07	2.87	0.00	4.57	0.77	0.15	13.84	0.00	0.25	0.16
1164	1169	Pinocarvone	30460-92-5	$C_{10}H_{14}O$	0.00	0.00	4.33	0.00	1.83	0.00	0.00	0.55	0.00	4.60	0.00	3.36	0.00	0.00	3.62	0.00	0.00	1.00	1.14	0.43	0.00	0.19	0.19
1177	1186	Terpinen-4-ol	562-74-3	$C_{10}H_{18}O$	0.09	0.15	1.08	0.12	0.40	0.24	1.03	0.24	0.11	0.92	0.08	0.50	0.25	0.18	3.82	0.08	0.29	0.31	2.40	1.28	0.83	0.61	0.66
1188	1199	α-Terpineol	98-55-5	$C_{10}H_{18}O$	0.00	0.30	0.00	0.14	0.00	0.14	0.00	0.25	0.00	0.00	0.00	2.54	0.09	0.44	2.97	0.09	0.46	0.23	4.39	2.51	1.82	0.55	0.20
1235	1233	3-Hexenyl isovalerate	35154-45-1	$C_{11}H_{20}O_2$	0.00	0.25	0.00	0.18	0.08	0.13	0.00	0.19	0.09	0.00	0.04	0.39	0.10	0.06	0.14	0.22	0.00	0.58	0.10	0.00	0.73	0.19	0.20
1352	1358	α-Longipinene	5989-08-02	$C_{15}H_{24}$	0.07	0.32	0.00	0.18	0.45	0.00	0.00	1.12	0.00	0.30	0.00	0.26	0.00	0.24	0.00	0.10	0.00	1.11	0.00	0.62	0.37	0.30	0.35
1376	1382	α-Copaene	3856-25-5	$C_{15}H_{24}$	0.13	0.27	0.46	0.20	0.18	0.30	0.26	0.62	0.53	0.72	0.36	0.16	0.07	0.18	0.22	0.25	0.45	0.35	0.50	0.43	0.35	0.40	0.79
-	1390	Benzyl isovalerate	103-38-8	$C_{12}H_{16}O_2$	0.38	0.38	0.79	1.08	2.25	0.00	0.98	0.98	0.29	0.71	0.49	0.33	0.65	0.82	0.61	2.46	0.79	1.49	1.55	1.25	1.28	0.37	0.48
1388	1393	β-Cubebene	13744-15-5	$C_{15}H_{24}$	2.71	1.69	6.00	4.68	10.89	6.79	8.01	3.67	2.63	13.03	1.84	1.74	1.34	2.20	6.03	2.43	2.09	2.54	2.68	3.74	3.26	5.27	2.55
1419	1428	β-Caryophyllene	87-44-5	$C_{15}H_{24}$	1.29	1.41	3.27	4.24	5.82	6.41	10.78	2.76	2.18	9.37	2.56	1.51	1.27	2.04	4.58	4.12	2.03	2.83	2.79	2.05	1.10	4.02	2.51
1456	1456	β-Farnesene	18794-84-8	$C_{15}H_{24}$	0.64	1.31	0.24	1.19	0.22	1.24	0.00	1.05	1.47	1.62	0.64	0.58	0.35	0.74	0.27	1.31	1.56	2.52	1.96	2.19	1.81	1.79	1.95
1454	1464	α-Humulene	6753-98-6	$C_{15}H_{24}$	0.14	0.16	0.53	0.42	0.68	0.59	1.02	0.30	0.00	0.80	0.23	0.16	0.12	0.22	0.44	0.46	0.22	0.33	0.36	0.24	0.00	0.36	0.27
1477	1481	β-Chamigrene	18431-82-8	$C_{15}H_{24}$	0.04	0.00	0.29	0.25	0.00	0.00	0.00	0.20	0.23	0.49	0.09	0.15	0.00	0.00	0.56	0.00	0.10	0.35	0.16	0.00	0.36	0.00	0.22
1490	1497	β-Selinene	17066-67-0	$C_{15}H_{24}$	2.43	1.51	20.05	0.65	8.54	3.58	26.97	0.96	2.65	5.76	4.09	1.52	1.24	0.26	0.98	1.88	6.96	3.60	8.92	6.04	6.90	1.20	1.26
1436	1503	γ-Elemene	3242-08-08	$C_{15}H_{24}$	0.23	0.00	0.66	0.34	0.05	0.41	0.00	0.04	0.09	1.25	0.00	0.08	0.05	0.16	0.46	0.22	0.00	0.00	0.16	0.20	0.20	0.46	0.00
1515	1506	Butylated hydroxytoluene	128-37-0	$C_{15}H_{24}O$	0.32	0.44	0.30	0.54	0.67	0.45	0.88	0.45	0.48	0.36	0.50	0.35	0.22	0.26	0.27	0.28	0.25	0.19	0.26	0.29	0.29	0.25	0.27
1523	1523	δ-Cadinene	483-76-1	$C_{15}H_{24}$	0.00	0.00	0.04	0.00	0.13	0.03	0.00	0.04	0.00	0.00	0.00	0.00	0.00	0.06	0.00	0.03	0.25	0.00	0.02	0.00	0.00	0.04	0.09
1583	1592	Caryophyllene oxide	1139-30-6	$C_{15}H_{24}O$	2.34	1.19	1.73	0.63	3.17	3.55	13.17	0.13	1.00	3.17	0.73	2.71	0.44	1.40	1.63	7.82	2.88	3.51	1.22	3.26	0.64	2.77	3.78
1646	1646	α-Muurolol	19435-97-3	$C_{15}H_{26}O$	0.95	0.69	3.13	0.51	5.12	1.58	0.00	0.00	1.33	0.00	0.38	0.00	0.00	1.05	0.00	3.26	1.51	0.00	0.80	0.91	0.53	0.00	0.00
1651	1659	Vulgarone B	64180-68-3	$C_{15}H_{22}O$	0.35	0.72	0.00	0.14	0.00	0.00	0.00	0.18	0.00	0.00	0.00	0.00	0.00	0.44	0.00	0.21	0.00	0.60	0.00	0.92	0.60	0.00	0.00
1761	1756	Lanceol	10067-29-5	$C_{15}H_{24}O$	0.72	0.56	1.43	0.00	3.34	0.81	2.49	0.00	1.63	0.64	0.17	0.33	0.18	0.53	0.66	1.93	0.44	1.29	0.55	0.45	0.51	0.41	0.95
Total					93.24	94.20	69.60	92.51	77.32	92.08	79.44	96.65	88.60	91.59	91.59	95.37	96.65	93.42	89.12	91.42	91.40	93.86	81.88	95.44	87.98	85.62	90.86

RI: retention indices; RI[a]: comparison retention indices with those reported in the literature (Adams, 2007); RI[b]: calculated retention indices which relative to n-alkanes (C_8-C_{20}) on the VF-5MS column condition.

(continued)

Chemical information					*A. annua* individuals																						
RI[a]	RI[b]	Name	Cas no.	Formula	AA28	AA29	AA30	AA31	AA32	AA33	AA34	AA35	AA36	AA37	AA38	AA39	AA40	AA41	AA42	AA43	AA44	AA45	AA46	AA47	AA48	AA49	AA50
908	903	Santolina triene	2153-66-4	$C_{10}H_{16}$	0.95	0.54	0.00	0.00	0.29	0.33	0.27	0.92	0.84	0.93	0.42	0.77	0.56	0.00	0.00	0.00	0.00	0.00	0.00	0.00	0.00	0.00	0.00
939	939	α-Pinene	80-56-8	$C_{10}H_{16}$	0.69	14.77	1.85	13.33	0.18	0.00	0.00	0.23	0.00	0.16	0.08	0.06	0.08	0.00	0.00	0.00	0.00	0.90	0.08	0.35	0.06	0.02	0.02
954	956	Camphene	79-92-5	$C_{10}H_{16}$	0.13	2.62	4.20	2.41	0.00	0.00	0.05	0.22	0.00	0.06	0.00	0.00	0.00	0.00	0.00	0.00	0.00	0.05	0.20	0.40	0.00	0.05	0.00
979	982	β-Pinene	127-91-3	$C_{10}H_{16}$	1.81	8.89	19.23	14.97	1.80	0.32	0.46	1.19	0.62	1.16	1.53	0.75	0.88	0.00	0.14	0.00	0.16	0.23	0.47	2.12	0.42	0.15	0.25
999	997	Yomogi alcohol	26127-98-0	$C_{10}H_{18}O$	1.65	3.17	0.20	0.06	1.55	1.59	1.12	1.63	1.23	1.87	1.33	1.29	0.95	0.00	0.00	0.00	0.00	0.05	0.02	0.15	0.09	0.06	0.44
1017	1020	α-Terpinene	99-86-5	$C_{10}H_{16}$	0.10	0.64	0.53	0.69	0.18	0.00	0.03	0.06	0.02	0.07	0.10	0.00	0.06	0.00	0.00	0.00	0.00	0.03	0.06	0.15	0.22	0.02	0.00
1094	1029	p-Cymene	99-87-6	$C_{10}H_{14}$	0.05	0.16	0.15	0.18	0.06	0.00	0.05	0.00	0.00	0.06	0.10	0.00	0.06	0.07	0.05	0.00	0.04	0.10	0.13	0.26	0.38	0.07	0.04
1029	1034	Limonene	138-86-3	$C_{10}H_{16}$	0.00	0.42	0.50	0.49	0.04	0.00	0.00	0.00	0.00	0.00	0.00	0.00	0.00	0.00	0.00	0.00	0.20	0.10	0.12	0.05	0.00	0.00	0.00
1131	1038	Eucalyptol	470-82-6	$C_{10}H_{18}O$	5.52	16.49	12.22	15.07	6.83	2.37	2.76	5.15	2.83	4.97	8.88	3.64	3.50	0.94	0.61	0.10	0.72	0.29	3.37	7.58	15.23	0.94	0.42
1062	1064	Artemisyl ketone	546-49-6	$C_{10}H_{16}O$	58.20	0.00	0.00	0.00	59.91	74.89	71.97	49.90	66.67	56.62	64.84	70.34	58.82	0.00	0.00	0.20	0.00	0.93	0.00	3.41	2.54	1.77	12.83
1070	1075	Sabinene hydrate	15537-55-0	$C_{10}H_{18}O$	0.00	0.07	0.08	0.00	0.00	0.00	0.00	0.00	0.00	0.00	0.08	0.00	0.00	0.00	0.00	0.00	0.00	0.00	0.00	0.00	0.00	0.00	0.00
1083	1083	Artemisia alcohol	27644-04-8	$C_{10}H_{18}O$	1.73	6.44	0.29	0.03	1.40	2.48	3.79	2.67	4.61	2.63	2.32	3.66	1.71	0.09	0.00	0.00	0.03	0.05	0.01	0.08	0.06	0.06	0.21
1114	1114	3-Isopentenyl isovalerate	54410-94-5	$C_{10}H_{18}O_2$	0.47	0.00	0.00	0.04	0.03	0.10	0.12	0.12	0.14	0.06	0.08	0.25	0.09	0.05	0.02	0.00	0.04	0.04	0.04	0.05	0.14	0.03	0.04
1139	1148	Pinocarveol	547-61-5	$C_{10}H_{16}O$	0.12	2.21	0.35	2.15	0.00	0.00	0.00	0.16	0.00	0.00	0.00	0.00	0.00	0.12	0.00	0.00	0.00	1.06	0.16	0.14	0.17	0.11	0.00
1146	1155	Camphor	76-22-2	$C_{10}H_{16}O$	0.77	9.86	25.05	14.30	0.19	0.05	1.07	1.98	0.00	0.60	0.47	0.00	0.19	0.03	0.04	0.10	0.04	3.42	5.18	2.04	0.07	4.40	0.00
1164	1169	Pinocarvone	30460-92-5	$C_{10}H_{14}O$	0.22	0.00	0.00	2.41	0.15	0.06	0.22	0.00	0.15	0.00	0.00	0.00	0.00	0.00	0.00	0.00	0.00	1.23	0.00	0.00	0.00	0.00	0.00
1177	1186	Terpinen-4-ol	562-74-3	$C_{10}H_{18}O$	0.42	2.39	2.31	3.19	0.43	0.39	0.34	0.59	0.24	0.40	0.66	0.18	0.30	0.26	0.05	0.03	0.12	0.28	0.38	0.55	1.44	0.14	0.07
1188	1199	α-Terpineol	98-55-5	$C_{10}H_{18}O$	0.28	1.91	1.63	2.33	0.36	0.00	0.11	0.69	0.12	0.55	0.64	0.25	0.27	0.07	0.00	0.00	0.00	0.00	0.05	0.16	0.31	0.00	0.00
1235	1233	3-Hexenyl isovalerate	35154-45-1	$C_{11}H_{20}O_2$	0.16	0.26	0.27	0.11	0.00	0.00	0.00	0.05	0.24	0.21	0.08	0.06	0.08	0.05	0.02	0.00	0.06	0.17	0.08	0.13	0.29	0.05	0.12
1352	1358	α-Longipinene	5989-08-02	$C_{15}H_{24}$	0.56	0.00	0.00	0.00	0.00	0.13	0.69	0.15	0.21	0.59	0.05	0.23	0.25	1.73	0.15	0.02	0.42	0.46	0.56	0.21	0.33	0.14	0.99
1376	1382	α-Copaene	3856-25-5	$C_{15}H_{24}$	0.73	0.41	0.33	0.07	0.43	0.39	0.34	0.47	0.14	0.29	0.21	0.29	0.26	0.76	0.27	0.18	0.76	0.33	0.57	0.43	0.70	0.31	0.63
-	1390	Benzyl isovalerate	103-38-8	$C_{12}H_{16}O_2$	0.42	0.55	0.47	0.39	1.24	0.38	0.64	1.19	0.24	0.68	0.81	0.95	0.47	3.85	1.93	1.54	3.82	2.35	3.45	2.90	2.98	2.12	1.83
1388	1393	β-Cubebene	13744-15-5	$C_{15}H_{24}$	4.15	3.77	4.35	3.03	4.70	2.45	2.80	3.05	2.20	5.49	3.19	3.10	3.74	4.96	10.39	5.37	12.64	16.22	7.75	13.90	15.73	13.52	12.57
1419	1428	β-Caryophyllene	87-44-5	$C_{15}H_{24}$	4.89	2.50	5.38	5.20	2.43	0.82	0.88	2.46	1.56	2.08	0.99	1.78	2.52	4.10	5.54	5.09	5.30	9.01	6.19	6.37	9.74	6.23	13.58
1456	1456	β-Farnesene	18794-84-8	$C_{15}H_{24}$	2.22	0.25	0.40	0.28	1.03	1.42	1.32	2.24	1.53	2.91	0.99	1.51	1.61	0.12	0.21	0.17	0.42	0.48	0.25	0.52	1.11	0.20	0.62
1454	1464	α-Humulene	6753-98-6	$C_{15}H_{24}$	0.46	0.21	0.45	0.37	0.33	0.11	0.19	0.30	0.22	0.28	0.00	0.19	0.31	0.00	0.00	0.78	0.00	1.13	0.00	0.00	1.30	0.77	1.40
1477	1481	β-Chamigrene	18431-82-8	$C_{15}H_{24}$	0.16	0.00	0.00	0.00	0.24	0.15	0.13	0.12	0.09	0.19	0.00	0.19	0.30	0.00	0.00	0.00	0.00	0.00	0.00	0.00	0.00	0.00	0.00
1490	1497	β-Selinene	17066-67-0	$C_{15}H_{24}$	2.12	0.23	0.54	0.36	2.31	0.09	0.51	10.41	4.28	5.52	0.21	0.29	0.39	20.78	34.40	22.89	32.99	20.84	38.33	8.35	1.05	46.29	21.64
1436	1503	γ-Elemene	3242-08-08	$C_{15}H_{24}$	0.45	0.19	2.25	0.31	0.28	0.14	0.20	0.18	0.23	0.39	0.14	0.14	0.30	0.60	0.58	0.24	1.12	1.52	0.80	0.78	1.36	0.81	0.85
1515	1506	Butylated hydroxytoluene	128-37-0	$C_{15}H_{24}O$	0.30	0.22	0.23	0.23	0.28	0.25	0.32	0.29	0.30	0.31	0.29	0.37	0.32	0.37	0.19	0.23	0.18	0.21	0.17	0.29	0.20	0.18	0.17
1523	1523	δ-Cadinene	483-76-1	$C_{15}H_{24}$	0.07	0.04	0.00	1.18	0.02	0.07	0.02	0.16	0.02	0.04	0.02	0.00	0.02	0.59	0.44	0.16	0.44	0.67	0.47	0.35	0.45	0.42	0.59
1583	1592	Caryophyllene oxide	1139-30-6	$C_{15}H_{24}O$	2.04	1.06	1.21	0.96	4.65	1.29	0.77	1.16	1.76	2.71	2.56	2.77	5.47	11.72	1.57	12.11	4.35	5.37	4.09	1.58	11.71	1.32	2.83
1646	1646	α-Muurolol	19435-97-3	$C_{15}H_{26}O$	0.82	0.00	0.00	0.00	0.00	0.00	0.54	0.00	1.49	1.41	1.05	1.12	2.69	15.38	9.91	9.90	9.12	6.41	6.51	8.65	9.62	3.50	6.02
1651	1659	Vulgarone B	64180-68-3	$C_{15}H_{22}O$	0.67	0.00	0.00	0.00	0.00	0.18	0.17	0.36	0.37	0.51	0.00	0.49	1.09	0.00	0.00	0.00	0.00	0.00	0.00	0.00	0.00	0.00	0.00
1761	1756	Lanceol	10067-29-5	$C_{15}H_{24}O$	0.37	0.23	0.18	0.00	0.62	0.61	0.18	1.37	0.09	0.73	0.91	0.72	1.34	5.44	3.29	3.98	3.80	2.89	0.00	4.12	2.40	1.86	1.78
Total					93.70	80.50	84.65	84.14	91.96	91.06	92.06	89.47	92.44	94.48	93.03	95.39	88.63	72.08	69.80	63.09	76.77	76.82	79.49	66.07	80.10	85.54	79.94

RI: retention indices; RI[a]: comparison retention indices with those reported in the literature (Adams, 2007); RI[b]: calculated retention indices which relative to n-alkanes (C_8-C_{20}) on the VF-5MS column condition.

(continued)

Chemical information					*A. annua* individuals																						
RI[a]	RI[b]	Name	Cas no.	Formula	AA51	AA52	AA53	AA54	AA55	AA56	AA57	AA58	AA59	AA60	AA61	AA62	AA63	AA64	AA65	AA66	AA67	AA68	AA69	AA70	AA71	AA72	AA74
908	903	Santolina triene	2153-66-4	$C_{10}H_{16}$	0.00	0.00	0.00	0.00	0.00	0.00	0.00	0.00	0.00	0.00	0.29	0.38	0.00	0.14	0.08	0.20	0.17	0.26	0.22	0.38	0.66	0.00	0.53
939	939	α-Pinene	80-56-8	$C_{10}H_{16}$	0.38	0.00	0.11	0.45	0.05	8.08	0.00	3.16	6.01	0.00	4.78	3.78	1.47	2.05	1.80	0.80	0.00	0.48	0.28	0.31	0.62	0.00	0.00
954	956	Camphene	79-92-5	$C_{10}H_{16}$	0.14	0.00	0.25	0.40	0.03	0.27	0.00	0.10	0.22	0.00	0.16	0.24	0.77	1.48	0.08	1.24	0.00	0.48	0.00	0.00	0.02	0.00	0.07
979	982	β-Pinene	127-91-3	$C_{10}H_{16}$	1.28	0.05	0.11	0.29	0.11	0.77	0.34	0.89	0.75	0.00	1.55	0.70	2.92	6.05	0.42	4.34	1.35	1.38	0.53	0.70	4.53	0.49	0.72
999	997	Yomogi alcohol	26127-98-0	$C_{10}H_{18}O$	0.17	0.07	0.05	0.02	0.25	0.12	0.03	0.13	0.10	0.07	0.72	0.72	0.53	0.42	1.12	0.49	0.77	0.60	0.52	0.90	0.69	3.26	1.36
1017	1020	α-Terpinene	99-86-5	$C_{10}H_{16}$	0.05	0.02	0.02	0.06	0.03	0.11	0.05	0.06	0.09	0.00	0.13	0.05	0.16	0.21	0.04	0.28	0.12	0.21	0.19	0.15	0.27	0.06	0.05
1094	1029	p-Cymene	99-87-6	$C_{10}H_{14}$	0.09	0.03	0.04	0.08	0.11	0.18	0.08	0.12	0.18	0.00	0.07	0.00	0.07	0.07	0.02	0.08	0.05	0.00	0.07	0.05	0.07	0.00	0.00
1029	1034	Limonene	138-86-3	$C_{10}H_{16}$	0.18	0.00	0.00	0.05	0.02	0.40	0.03	0.03	0.16	0.00	0.15	0.11	0.17	0.11	0.00	0.09	0.11	0.11	0.13	0.08	0.21	0.00	0.00
1131	1038	Eucalyptol	470-82-6	$C_{10}H_{18}O$	1.94	0.79	0.76	1.82	2.48	2.09	2.06	2.14	3.62	0.07	10.24	4.85	15.55	14.93	1.63	18.31	9.86	14.11	18.00	12.53	24.30	6.15	3.79
1062	1064	Artemisyl ketone	546-49-6	$C_{10}H_{16}O$	10.48	1.55	1.67	0.00	17.43	2.76	0.00	3.91	1.17	1.72	49.43	65.77	43.04	25.99	68.76	31.70	56.48	42.46	47.66	59.31	27.43	60.77	54.57
1070	1075	Sabinene hydrate	15537-55-0	$C_{10}H_{18}O$	0.00	0.00	0.00	0.00	0.00	0.00	0.00	0.00	0.00	0.00	0.22	0.19	0.32	0.32	0.08	0.30	0.21	0.29	1.12	0.12	0.47	0.23	0.00
1083	1083	Artemisia alcohol	27644-04-8	$C_{10}H_{18}O$	0.08	0.07	0.03	0.00	0.10	0.11	0.00	0.12	0.09	0.05	2.07	2.69	1.86	1.65	1.87	1.31	2.11	1.49	2.10	2.07	2.89	11.98	5.08
1114	1114	3-Isopentenyl isovalerate	54410-94-5	$C_{10}H_{18}O_2$	0.03	0.03	0.03	0.02	0.02	0.03	0.02	0.02	0.02	0.04	0.17	0.11	0.07	0.09	0.07	0.08	0.10	0.09	0.11	0.13	0.17	0.26	0.14
1139	1148	Pinocarveol	547-61-5	$C_{10}H_{16}O$	0.10	0.08	0.00	0.09	0.00	0.36	0.00	0.27	1.26	0.00	1.16	1.76	0.43	0.86	1.04	0.29	0.00	0.25	0.00	0.10	1.33	0.00	0.00
1146	1155	Camphor	76-22-2	$C_{10}H_{16}O$	0.28	0.12	2.19	2.07	0.40	1.11	0.07	0.76	2.73	0.16	1.32	1.74	4.65	8.39	0.89	5.81	0.00	2.85	0.00	0.10	0.11	0.00	0.14
1164	1169	Pinocarvone	30460-92-5	$C_{10}H_{14}O$	0.00	0.00	0.00	0.00	0.00	0.00	0.00	0.00	0.00	0.00	1.34	1.29	0.61	1.00	0.98	0.40	0.32	0.32	0.29	0.16	0.16	0.00	0.00
1177	1186	Terpinen-4-ol	562-74-3	$C_{10}H_{18}O$	0.14	0.17	0.10	0.19	0.10	0.29	0.19	0.36	0.52	0.02	0.63	0.25	0.71	0.81	0.21	1.11	0.44	0.91	0.82	0.63	1.11	0.25	0.18
1188	1199	α-Terpineol	98-55-5	$C_{10}H_{18}O$	0.00	0.02	0.04	0.03	0.00	0.00	0.02	0.00	0.10	0.00	0.65	0.22	1.04	1.37	0.00	1.68	0.35	0.84	0.67	0.65	1.89	0.26	0.14
1235	1233	3-Hexenyl isovalerate	35154-45-1	$C_{11}H_{20}O_2$	0.03	0.04	0.15	0.08	0.00	0.13	0.08	0.15	0.19	0.07	0.17	0.16	0.15	0.30	0.10	0.38	0.17	0.15	0.20	0.08	0.22	0.25	0.11
1352	1358	α-Longipinene	5989-08-02	$C_{15}H_{24}$	0.00	0.99	1.70	0.38	0.04	1.05	0.41	1.15	0.61	0.07	0.58	0.33	0.43	0.62	1.16	0.74	0.40	0.77	0.66	0.62	1.20	0.81	0.98
1376	1382	α-Copaene	3856-25-5	$C_{15}H_{24}$	2.90	0.26	0.44	0.41	0.19	0.42	0.84	0.53	0.42	0.75	0.99	0.22	0.38	0.35	0.40	0.64	0.34	0.62	0.46	0.44	0.48	0.33	1.04
-	1390	Benzyl isovalerate	103-38-8	$C_{12}H_{16}O_2$	2.95	1.72	3.16	1.69	1.26	3.51	1.53	3.67	3.11	0.96	0.94	0.64	1.16	0.67	1.16	0.81	1.01	1.19	0.77	0.98	1.00	0.82	0.75
1388	1393	β-Cubebene	13744-15-5	$C_{15}H_{24}$	21.37	12.59	11.86	14.76	22.52	9.50	17.58	9.00	8.35	16.25	2.46	1.78	2.94	3.37	2.59	3.56	3.42	3.87	3.43	2.05	2.81	3.30	8.97
1419	1428	β-Caryophyllene	87-44-5	$C_{15}H_{24}$	6.22	4.10	8.83	6.18	5.45	5.53	9.74	7.17	7.19	13.14	2.39	1.26	2.43	3.35	2.20	2.37	2.45	3.09	2.95	2.08	2.70	1.97	5.78
1456	1456	β-Farnesene	18794-84-8	$C_{15}H_{24}$	1.40	0.49	0.26	0.45	1.22	0.29	0.97	0.26	0.29	0.44	1.61	1.02	1.36	1.40	1.55	1.64	1.43	1.57	1.49	1.29	1.63	0.86	3.26
1454	1464	α-Humulene	6753-98-6	$C_{15}H_{24}$	0.00	0.00	0.00	0.00	0.77	0.00	0.00	0.00	0.00	1.24	0.24	0.00	0.28	0.30	0.20	0.30	0.00	0.34	0.30	0.23	0.29	0.20	0.51
1477	1481	β-Chamigrene	18431-82-8	$C_{15}H_{24}$	0.00	0.00	0.00	0.00	0.00	0.00	0.00	0.00	0.00	0.00	0.13	0.00	0.00	0.12	0.00	0.00	0.00	0.00	0.00	0.13	0.00	0.14	0.00
1490	1497	β-Selinene	17066-67-0	$C_{15}H_{24}$	1.87	24.10	34.79	29.12	0.45	40.71	6.75	25.78	30.38	34.84	7.24	4.99	6.52	7.51	6.29	9.07	10.20	9.44	9.42	7.87	9.05	0.13	0.27
1436	1503	γ-Elemene	3242-08-08	$C_{15}H_{24}$	1.95	1.15	0.87	1.39	1.12	0.77	0.98	0.75	0.44	0.84	0.12	0.00	0.16	0.16	0.11	0.20	0.19	0.21	0.18	0.10	0.17	0.19	0.62
1515	1506	Butylated hydroxytoluene	128-37-0	$C_{15}H_{24}O$	0.29	0.18	0.15	0.17	0.20	0.15	0.00	0.18	0.13	0.13	0.24	0.31	0.25	0.29	0.26	0.25	0.26	0.26	0.28	0.26	0.25	0.29	0.29
1523	1523	δ-Cadinene	483-76-1	$C_{15}H_{24}$	0.33	0.45	0.34	0.57	0.42	0.28	0.46	0.60	0.38	0.40	0.03	0.00	0.04	0.00	0.00	0.04	0.04	0.05	0.03	0.03	0.10	0.03	0.17
1583	1592	Caryophyllene oxide	1139-30-6	$C_{15}H_{24}O$	0.00	2.28	1.90	1.96	3.68	2.42	3.33	6.44	4.11	3.89	0.22	0.46	1.00	0.76	0.51	0.77	0.93	0.53	0.45	0.00	0.46	0.73	1.52
1646	1646	α-Muurolol	19435-97-3	$C_{15}H_{26}O$	9.73	15.47	6.36	11.27	4.05	3.75	14.02	6.79	5.82	4.61	0.00	0.13	0.48	0.00	0.00	0.00	0.51	0.00	0.41	0.00	0.00	0.32	0.00
1651	1659	Vulgarone B	64180-68-3	$C_{15}H_{22}O$	0.00	0.00	0.00	0.00	0.00	0.00	0.00	0.00	0.00	0.00	0.38	0.14	0.16	0.20	0.66	0.22	0.14	0.41	0.17	0.16	0.33	0.15	0.25
1761	1756	Lanceol	10067-29-5	$C_{15}H_{24}O$	3.29	5.43	3.03	2.51	3.96	2.19	3.94	0.43	0.00	0.48	0.00	0.00	0.00	0.00	0.00	0.00	0.15	0.17	0.19	0.00	0.00	0.00	0.00
		Total			67.67	72.25	79.24	76.51	66.46	87.38	63.52	74.97	78.44	80.24	92.82	96.29	92.11	85.34	96.28	89.50	94.08	89.80	94.10	94.69	87.62	94.23	91.29

RI: retention indices; RI[a]: comparison retention indices with those reported in the literature (Adams, 2007); RI[b]: calculated retention indices which relative to n-alkanes (C_8-C_{20}) on the VF-5MS column condition.

(continued)

Chemical information					*A. annua* individuals																						
RI[a]	RI[b]	Name	Cas no.	Formula	AA75	AA78	AA80	AA82	AA83	AA84	AA85	AA86	AA87	AA88	AA89	AA90	AA91	AA92	AA93	AA94	AA95	AA96	AA97	AA98	AA99	AA100	AA101
908	903	Santolina triene	2153-66-4	$C_{10}H_{16}$	0.26	0.22	0.31	0.06	0.17	0.22	0.17	0.20	0.38	0.39	0.56	0.48	0.64	0.88	0.95	0.61	0.77	1.02	0.49	0.82	0.89	1.26	0.71
939	939	α-Pinene	80-56-8	$C_{10}H_{16}$	0.16	0.00	0.52	0.05	1.28	0.82	3.08	0.14	0.31	1.83	3.21	3.10	2.24	0.68	2.05	6.84	4.45	1.69	1.16	2.18	0.74	0.61	1.56
954	956	Camphene	79-92-5	$C_{10}H_{16}$	0.00	0.00	2.34	0.00	0.05	0.00	0.57	0.00	0.00	0.05	0.16	0.00	0.00	0.00	0.06	0.23	0.13	0.04	0.03	0.17	0.00	0.17	0.23
979	982	β-Pinene	127-91-3	$C_{10}H_{16}$	0.40	1.28	8.48	0.33	0.27	1.73	1.69	0.89	0.54	1.07	0.76	1.15	0.53	2.11	0.56	2.16	1.66	2.17	0.72	0.76	2.94	0.73	2.10
999	997	Yomogi alcohol	26127-98-0	$C_{10}H_{18}O$	1.64	2.52	0.73	0.89	0.80	0.33	0.48	1.26	0.98	0.63	0.58	0.73	0.92	0.68	0.74	0.72	0.84	0.61	0.87	0.58	0.63	0.66	0.51
1017	1020	α-Terpinene	99-86-5	$C_{10}H_{16}$	0.05	0.05	0.13	0.11	0.04	0.28	0.27	0.07	0.20	0.12	0.03	0.07	0.00	0.17	0.05	0.09	0.07	0.07	0.08	0.08	0.08	0.38	0.05
1094	1029	p-Cymene	99-87-6	$C_{10}H_{14}$	0.01	0.02	0.00	0.11	0.03	0.07	0.09	0.02	0.13	0.08	0.06	0.03	0.00	0.05	0.00	0.11	0.00	0.00	0.04	0.04	0.00	0.17	0.06
1029	1034	Limonene	138-86-3	$C_{10}H_{16}$	0.00	0.08	0.30	0.11	0.14	0.17	0.14	0.00	0.14	0.16	0.00	0.00	0.13	0.14	0.08	0.16	0.18	0.09	0.16	0.17	0.08	0.00	0.08
1131	1038	Eucalyptol	470-82-6	$C_{10}H_{18}O$	4.92	4.41	8.87	9.98	4.24	31.00	19.66	4.46	12.14	11.55	2.87	5.21	2.13	12.36	7.10	4.59	5.87	9.65	9.64	10.17	10.22	2.70	6.86
1062	1064	Artemisyl ketone	546-49-6	$C_{10}H_{16}O$	68.44	56.34	44.18	67.16	65.35	25.47	27.99	60.59	51.97	51.53	55.49	63.85	71.08	54.12	68.11	54.88	59.94	57.31	66.11	50.47	52.93	75.11	63.46
1070	1075	Sabinene hydrate	15537-55-0	$C_{10}H_{18}O$	0.29	0.14	0.13	0.47	0.19	1.16	0.83	0.00	0.41	0.61	0.17	0.17	0.00	0.32	0.34	0.51	0.35	0.44	0.20	0.38	0.57	0.00	0.11
1083	1083	Artemisia alcohol	27644-04-8	$C_{10}H_{18}O$	6.93	9.23	3.29	2.04	1.90	1.23	1.86	3.50	2.08	2.98	2.77	3.32	3.27	2.15	3.52	3.10	3.47	3.34	3.39	2.34	2.82	2.59	3.35
1114	1114	3-Isopentenyl isovalerate	54410-94-5	$C_{10}H_{18}O_2$	0.15	0.11	0.14	0.14	0.10	0.11	0.18	0.13	0.09	0.19	0.21	0.08	0.14	0.16	0.10	0.09	0.11	0.09	0.00	0.15	0.14	0.00	0.11
1139	1148	Pinocarveol	547-61-5	$C_{10}H_{16}O$	0.22	0.00	0.29	0.00	0.33	0.21	0.83	0.00	0.00	0.54	0.82	0.61	0.39	0.20	0.36	1.51	0.78	0.35	0.23	0.21	0.09	0.00	0.25
1146	1155	Camphor	76-22-2	$C_{10}H_{16}O$	0.00	0.12	10.12	0.15	0.41	0.00	3.23	0.15	0.04	0.39	1.91	1.00	0.77	0.08	0.77	1.93	1.10	0.53	0.32	1.06	0.07	1.11	1.43
1164	1169	Pinocarvone	30460-92-5	$C_{10}H_{14}O$	0.00	0.02	0.00	0.14	0.44	0.19	1.02	0.00	0.30	0.55	1.09	0.79	0.52	0.09	0.52	2.13	0.82	0.41	0.28	0.25	0.00	0.00	0.40
1177	1186	Terpinen-4-ol	562-74-3	$C_{10}H_{18}O$	0.23	0.40	0.48	0.54	0.16	1.15	1.15	0.26	0.84	0.48	0.18	0.27	0.08	0.48	0.20	0.34	0.25	0.29	0.39	0.25	0.29	0.34	0.20
1188	1199	α-Terpineol	98-55-5	$C_{10}H_{18}O$	0.22	0.07	0.46	0.00	0.00	2.26	0.95	0.12	0.61	0.58	0.17	0.30	0.01	1.25	0.65	0.50	0.60	0.94	0.89	0.77	0.94	0.14	0.45
1235	1233	3-Hexenyl isovalerate	35154-45-1	$C_{11}H_{20}O_2$	0.19	0.31	0.00	0.00	0.00	0.28	0.28	0.14	0.17	0.37	0.24	0.21	0.30	0.25	0.28	0.60	0.35	0.25	0.21	0.14	0.28	0.06	0.41
1352	1358	α-Longipinene	5989-08-02	$C_{15}H_{24}$	0.69	0.85	0.13	0.41	0.61	2.30	1.75	0.63	0.87	0.54	0.54	0.88	0.36	0.86	1.38	0.83	1.27	1.09	0.23	0.79	0.67	0.42	0.58
1376	1382	α-Copaene	3856-25-5	$C_{15}H_{24}$	0.30	0.53	0.10	0.28	0.76	0.44	0.56	0.70	0.60	0.37	0.42	0.18	0.22	0.33	0.18	0.27	0.22	0.24	0.17	0.40	0.41	0.13	0.15
-	1390	Benzyl isovalerate	103-38-8	$C_{12}H_{16}O_2$	0.74	0.76	0.42	0.81	1.13	1.08	1.14	0.57	1.33	0.97	1.25	1.03	0.74	0.95	0.80	0.99	1.14	1.00	0.72	1.03	0.80	1.00	0.68
1388	1393	β-Cubebene	13744-15-5	$C_{15}H_{24}$	3.09	5.62	2.50	3.48	2.72	3.52	4.35	7.08	3.45	1.96	4.73	2.69	0.00	3.64	1.78	3.48	3.03	2.58	2.16	4.81	3.42	3.90	2.64
1419	1428	β-Caryophyllene	87-44-5	$C_{15}H_{24}$	2.20	2.92	2.53	3.46	2.76	2.43	3.20	4.32	3.81	1.76	3.90	1.58	1.69	2.69	1.24	2.25	1.85	2.22	1.51	2.80	2.57	1.57	1.31
1456	1456	β-Farnesene	18794-84-8	$C_{15}H_{24}$	0.87	1.71	1.00	1.78	2.13	2.11	1.88	2.41	2.29	1.26	1.33	0.76	1.24	1.18	0.65	1.52	1.29	1.14	0.71	2.37	1.77	0.59	0.83
1454	1464	α-Humulene	6753-98-6	$C_{15}H_{24}$	0.20	0.29	0.22	0.31	0.00	0.31	0.34	0.40	0.38	0.25	0.49	0.19	0.19	0.34	0.16	0.27	0.22	0.24	0.16	0.34	0.30	0.00	0.00
1477	1481	β-Chamigrene	18431-82-8	$C_{15}H_{24}$	0.00	0.20	0.08	0.00	0.51	0.24	0.00	0.00	0.27	0.09	0.31	0.08	0.14	0.23	0.10	0.16	0.00	0.21	0.00	0.27	0.19	0.35	0.00
1490	1497	β-Selinene	17066-67-0	$C_{15}H_{24}$	0.14	0.24	1.21	1.48	8.01	6.59	9.26	0.33	5.95	10.83	6.05	5.53	5.35	5.43	2.71	3.20	3.98	5.46	4.09	7.68	8.62	0.69	6.02
1436	1503	γ-Elemene	3242-08-08	$C_{15}H_{24}$	0.17	0.38	0.14	0.24	0.13	0.19	0.27	0.49	0.19	0.00	0.31	0.16	0.00	0.24	0.06	0.22	0.24	0.14	0.13	0.30	0.26	0.00	0.15
1515	1506	Butylated hydroxytoluene	128-37-0	$C_{15}H_{24}O$	0.27	0.28	0.24	0.22	0.28	0.25	0.25	0.32	0.29	0.26	0.26	0.24	0.35	0.23	0.25	0.25	0.27	0.30	0.29	0.24	0.26	0.55	0.33
1523	1523	δ-Cadinene	483-76-1	$C_{15}H_{24}$	0.03	0.07	0.00	0.00	0.05	0.19	0.14	0.10	0.10	0.00	0.14	0.07	0.06	0.11	0.00	0.04	0.04	0.00	0.02	0.13	0.11	0.00	0.00
1583	1592	Caryophyllene oxide	1139-30-6	$C_{15}H_{24}O$	0.72	1.18	0.16	0.17	0.74	0.42	0.54	1.47	0.98	0.50	0.79	0.43	0.40	0.30	0.17	0.25	0.15	0.33	0.00	0.32	0.76	0.34	0.27
1646	1646	α-Muurolol	19435-97-3	$C_{15}H_{26}O$	0.32	0.00	0.11	0.00	0.57	0.59	0.65	0.00	0.72	0.34	1.06	0.00	0.00	0.00	0.00	0.32	0.00	0.00	0.00	0.00	0.49	0.00	0.00
1651	1659	Vulgarone B	64180-68-3	$C_{15}H_{22}O$	0.38	1.01	0.04	0.09	0.15	0.85	1.40	0.28	0.20	0.07	0.39	0.35	0.28	0.31	0.27	0.27	0.28	0.21	0.12	0.25	0.19	0.20	0.00
1761	1756	Lanceol	10067-29-5	$C_{15}H_{24}O$	0.00	0.00	0.00	0.00	0.00	0.19	0.00	0.31	0.18	0.00	0.00	0.00	0.09	0.00	0.00	0.00	0.00	0.00	0.00	0.21	0.00	0.00	0.00
Total					94.23	91.36	89.65	95.01	96.45	88.38	90.20	91.34	92.94	93.30	93.25	95.54	94.26	93.01	96.19	95.42	95.72	94.45	95.52	92.93	94.53	95.77	95.29

RI: retention indices; RI[a]: comparison retention indices with those reported in the literature (Adams, 2007); RI[b]: calculated retention indices which relative to n-alkanes (C_8-C_{20}) on the VF-5MS column condition.

(continued)

Chemical information					*A. annua* individuals										
RI[a]	RI[b]	Name	Cas no.	Formula	AA102	AA103	AA104	AA105	AA106	AA107	AA108	AA109	AA110	AA111	AA112
908	903	Santolina triene	2153-66-4	$C_{10}H_{16}$	0.47	0.82	0.74	2.15	0.44	0.48	0.31	0.96	0.36	0.75	0.82
939	939	α-Pinene	80-56-8	$C_{10}H_{16}$	1.17	1.87	3.92	1.81	0.00	0.72	4.88	0.48	4.18	0.81	1.64
954	956	Camphene	79-92-5	$C_{10}H_{16}$	0.00	0.00	0.14	0.04	0.00	0.10	0.19	0.31	0.10	0.02	0.09
979	982	β-Pinene	127-91-3	$C_{10}H_{16}$	1.06	1.91	1.85	6.05	0.93	1.37	0.58	1.21	0.56	1.96	0.75
999	997	Yomogi alcohol	26127-98-0	$C_{10}H_{18}O$	0.72	0.79	0.86	0.59	0.60	0.00	0.65	0.71	0.49	0.29	0.47
1017	1020	α-Terpinene	99-86-5	$C_{10}H_{16}$	0.04	0.04	0.05	0.08	0.15	0.03	0.00	0.15	0.04	0.08	0.02
1094	1029	p-Cymene	99-87-6	$C_{10}H_{14}$	0.00	0.00	0.04	0.00	0.09	0.00	0.10	0.09	0.03	0.04	0.00
1029	1034	Limonene	138-86-3	$C_{10}H_{16}$	0.12	0.15	0.18	0.17	0.00	0.04	0.11	0.09	0.04	0.10	0.09
1031	1038	Eucalyptol	470-82-6	$C_{10}H_{18}O$	4.08	6.07	4.53	9.80	4.36	4.91	2.57	7.80	3.01	20.00	5.60
1062	1064	Artemisia ketone	546-49-6	$C_{10}H_{16}O$	70.23	67.70	66.07	54.28	83.82	70.63	61.04	59.44	62.81	42.38	73.07
1070	1075	Sabinene hydrate	15537-55-0	$C_{10}H_{18}O$	0.07	0.15	0.17	0.24	0.00	0.08	0.21	0.00	0.07	0.49	0.13
1083	1083	Artemisia alcohol	27644-04-8	$C_{10}H_{18}O$	2.85	2.92	3.65	3.92	2.14	3.86	2.81	2.48	2.20	2.41	3.46
1114	1114	3-Isopentenyl isovalerate	54410-94-5	$C_{10}H_{18}O_2$	0.10	0.00	0.10	0.12	0.00	0.02	0.07	0.10	0.05	0.08	0.07
1139	1148	Pinocarveol	547-61-5	$C_{10}H_{16}O$	0.26	0.32	0.69	0.11	0.00	0.13	0.81	0.00	0.57	0.12	0.29
1146	1155	Camphor	76-22-2	$C_{10}H_{16}O$	0.46	0.48	1.34	0.23	0.23	0.97	2.10	1.74	1.38	0.10	0.73
1164	1169	Pinocarvone	30460-92-5	$C_{10}H_{14}O$	0.31	0.46	0.00	0.13	0.15	0.23	1.60	0.00	0.97	0.08	0.48
1177	1186	Terpinen-4-ol	562-74-3	$C_{10}H_{18}O$	0.16	0.24	0.23	0.18	0.00	0.17	0.22	0.24	0.14	0.36	0.12
1188	1199	α-Terpineol	98-55-5	$C_{10}H_{18}O$	0.30	0.44	0.27	0.88	0.05	0.34	0.16	0.66	0.20	1.18	0.33
1235	1233	3-Hexenyl isovalerate	35154-45-1	$C_{11}H_{20}O_2$	0.39	0.26	0.30	0.00	0.00	0.27	0.27	0.23	0.17	0.35	0.17
1352	1358	α-Longipinene	5989-08-02	$C_{15}H_{24}$	0.61	0.70	0.86	0.67	0.24	0.93	0.72	1.72	0.60	0.63	0.46
1376	1382	α-Copaene	3856-25-5	$C_{15}H_{24}$	0.23	0.16	0.15	0.28	0.09	0.17	0.34	0.45	0.32	0.31	0.13
-	1390	Benzyl isovalerate	103-38-8	$C_{12}H_{16}O_2$	0.87	0.80	0.78	0.84	0.20	0.80	1.11	0.36	0.72	0.99	0.48
1388	1393	β-Cubebene	13744-15-5	$C_{15}H_{24}$	2.77	1.85	2.10	0.00	0.00	2.09	4.04	5.49	5.32	3.16	0.00
1419	1428	β-Caryophyllene	87-44-5	$C_{15}H_{24}$	2.17	1.36	1.32	2.46	0.69	1.41	2.63	1.89	4.33	2.57	1.11
1456	1456	β-Farnesene	18794-84-8	$C_{15}H_{24}$	1.26	0.71	0.83	0.99	0.39	0.50	1.35	1.87	1.39	0.93	0.60
1454	1464	α-Humulene	6753-98-6	$C_{15}H_{24}$	0.24	0.15	0.14	0.24	0.00	0.15	0.29	0.00	0.37	0.37	0.00
1477	1481	β-Chamigrene	18431-82-8	$C_{15}H_{24}$	0.16	0.14	0.00	0.19	0.00	0.10	0.17	0.36	0.00	0.00	0.12
1490	1497	β-Selinene	17066-67-0	$C_{15}H_{24}$	4.84	4.16	3.36	5.68	1.85	5.15	4.74	2.46	5.17	9.74	3.23
1436	1503	γ-Elemene	3242-08-08	$C_{15}H_{24}$	0.15	0.10	0.13	0.00	0.09	0.11	0.25	0.49	0.28	0.16	0.09
1515	1506	Butylated hydroxytoluene	128-37-0	$C_{15}H_{24}O$	0.29	0.33	0.31	0.24	0.09	0.22	0.25	0.30	0.22	0.17	0.24
1523	1523	δ-Cadinene	483-76-1	$C_{15}H_{24}$	0.02	0.05	0.00	0.00	0.00	0.02	0.12	0.09	0.13	0.04	0.00
1583	1592	Caryophyllene oxide	1139-30-6	$C_{15}H_{24}O$	0.33	0.21	0.09	0.13	0.00	0.18	0.56	0.35	0.29	0.33	0.16
1646	1646	α-Muurolol	19435-97-3	$C_{15}H_{26}O$	0.00	0.23	0.00	0.00	0.00	0.00	0.00	0.00	0.00	0.00	0.00
1651	1659	Vulgarone B	64180-68-3	$C_{15}H_{22}O$	0.16	0.44	0.17	0.00	0.00	0.26	0.47	0.12	0.13	0.27	0.09
1761	1756	Lanceol	10067-29-5	$C_{15}H_{24}O$	0.00	0.08	0.00	0.00	0.00	0.00	0.00	0.25	0.00	0.00	0.00
Total					96.89	96.09	95.37	92.50	96.60	96.44	95.72	92.89	96.64	91.27	95.04

RI: retention indices; RI[a]: comparison retention indices with those reported in the literature (Adams, 2007); RI[b]: calculated retention indices which relative to n-alkanes (C_8-C_{20}) on the VF-5MS column condition.

Table S2. Correlation coefficients between 35 components of essential oils of Korean *Artemisia annua* individuals.

Code	C1	C2	C3	C4	C5	C6	C7	C8	C9	C10	C11	C12	C13	C14	C15	C16	C17	C18	C19	C20	C21	C22	C23	C24	C25	C26	C27	C28	C29	C30	C31	C32	C33	C34	C35
C1	1.000																																		
C2	.031	1.000																																	
C3	-.152	.357**	1.000																																
C4	.119	.443**	**.831****	1.000																															
C5	.314**	-.053	-.022	.012	1.000																														
C6	-.088	.548**	.593**	**.645****	-.008	1.000																													
C7	-.223*	.445**	.206*	.231*	-.199*	.569**	1.000																												
C8	.036	.549**	**.636****	**.706****	-.111	.594**	.245*	1.000																											
C9	.012	.184	.267**	.378**	-.032	**.672****	.213*	.487**	1.000																										
C10	.533**	-.276**	-.337**	-.216*	.499**	-.308**	-.425**	-.298**	-.150	1.000																									
C11	.022	.434**	.053	.172	-.120	.445**	.221*	.336**	.522**	-.077	1.000																								
C12	.414**	-.085	-.086	-.007	**.802****	-.120	-.320**	-.077	-.033	**.606****	-.008	1.000																							
C13	.168	-.131	-.130	-.088	.329**	-.025	-.004	-.071	.162	.280**	.130	.353**	1.000																						
C14	-.044	**.890****	.343**	.368**	-.076	.466**	.384**	.413**	.174	-.271**	.315**	-.101	-.093	1.000																					
C15	-.200*	.230*	**.936****	**.749****	-.109	.468**	.132	.570**	.194*	-.349**	.007	-.150	-.159	.246*	1.000																				
C16	-.033	**.797****	.193	.241*	-.178	.366**	.341**	.249*	.148	-.169	.363**	-.158	-.077	**.879****	.122	1.000																			
C17	-.211*	.569**	**.618****	**.640****	-.050	**.916****	.504**	**.605****	**.671****	-.405**	.513**	-.180	-.051	.493**	.549**	.407**	1.000																		
C18	.091	.392**	.400**	.576**	-.055	**.729****	.195*	**.611****	**.874****	-.201*	.512**	-.061	.043	.308**	.336**	.273**	**.746****	1.000																	
C19	.272**	.156	.016	.224*	.146	.071	-.046	.192	.264**	.154	.220*	.302**	.130	.117	-.029	.090	.069	.275**	1.000																
C20	.105	-.087	-.190	-.174	-.034	-.107	-.178	-.013	.156	.028	.144	.117	.183	-.064	-.199*	-.077	-.158	.027	.336**	1.000															
C21	-.246*	-.094	-.064	-.116	-.055	-.007	.090	-.019	.003	-.272**	-.103	-.190	.071	-.054	-.098	-.057	-.021	-.092	-.140	.055	1.000														
C22	-.427**	-.088	-.152	-.258**	-.427**	-.155	.306**	-.004	-.173	**-.616****	-.185	-.455**	-.104	-.097	-.087	-.155	-.114	-.208*	-.110	.220*	.336**	1.000													
C23	-.476**	-.042	-.073	-.208*	-.379**	-.098	.297**	-.139	-.270**	**-.678****	-.192	-.488**	-.262**	-.014	-.041	-.028	-.075	-.288**	-.273**	-.088	.462**	.559**	1.000												
C24	-.449**	.028	.034	-.088	-.394**	-.011	.306**	-.082	-.217*	**-.703****	-.102	-.505**	-.189	.051	.096	.026	.054	-.231*	-.249*	-.067	.331**	.495**	**.788****	1.000											
C25	.263**	-.217*	-.167	-.094	.403**	-.014	-.224*	-.103	.292**	.402**	.031	.258**	.409**	-.167	-.185	-.066	-.033	.162	.213*	.211*	.266**	-.336**	-.219*	-.276**	1.000										
C26	-.172	.041	.040	-.024	-.109	.065	.178	-.118	.012	-.297**	.067	-.199*	.037	.126	.091	.180	.153	-.028	-.076	-.165	.034	-.015	.377**	**.618****	.000	1.000									
C27	.376**	.301**	-.070	.072	.181	.227*	.083	.010	.101	.227*	.279**	.138	.088	.242*	-.146	.386**	.157	.165	.136	.047	-.010	-.272**	-.231*	-.223*	.330**	-.040	1.000								
C28	-.403**	-.060	-.095	-.251*	-.500**	-.215*	.033	-.049	-.222*	**-.659****	-.114	-.507**	-.261**	-.055	.020	-.095	-.151	-.217*	-.208*	.110	.080	**.661****	.469**	.549**	-.456**	.107	-.285**	1.000							
C29	-.426**	.002	.236*	.126	-.370**	.071	.331**	.101	-.182	**-.647****	-.199*	-.454**	-.225*	.029	.263**	.005	.093	-.158	-.183	-.078	.463**	.480**	**.800****	**.644****	-.213*	.259**	-.195*	.363**	1.000						
C30	.245*	-.011	-.017	-.014	.188	-.026	-.026	-.143	-.097	.235*	.097	.151	-.023	.030	.010	.087	-.006	-.067	-.069	-.162	-.101	-.235*	-.201*	-.100	.017	.120	.192	-.243*	-.322**	1.000					
C31	-.458**	.029	.046	.008	-.443**	.017	.239*	.069	-.181	**-.659****	-.243*	-.494**	-.191	.019	.115	-.050	.085	-.119	-.221*	.062	.212*	.590**	**.631****	**.614****	-.350**	.186	-.335**	.533**	.554**	-.364**	1.000				
C32	-.330**	-.083	-.086	-.183	-.228*	-.131	.229*	-.232*	-.207*	-.464**	-.112	-.372**	-.079	-.086	.002	-.091	.013	-.202*	-.228*	-.143	.057	.455**	.351**	.512**	-.250*	.423**	-.176	.329**	.255**	.165	.334**	1.000			
C33	-.458**	-.166	-.139	-.257**	-.457**	-.226*	.206*	-.192	-.321**	**-.657****	-.265**	-.507**	-.220*	-.153	-.119	-.178	-.176	-.307**	-.288**	.020	.312**	**.713****	**.697****	.553**	-.404**	.018	-.331**	.562**	**.611****	-.268**	**.677****	.523**	1.000		
C34	.187	-.131	-.080	-.052	.307**	-.054	-.293**	-.085	.185	.323**	.133	.298**	.300**	-.086	-.059	-.050	-.023	.133	.302**	.381**	-.035	-.232*	-.312**	-.339**	.506**	-.072	.175	-.285**	-.287**	.053	-.292**	-.181	-.312**	1.000	
C35	-.424**	-.137	-.129	-.236*	-.375**	-.213*	.173	-.219*	-.327**	**-.602****	-.215*	-.500**	-.245*	-.136	-.110	-.113	-.137	-.287**	-.330**	-.083	.229*	.597**	**.686****	.489**	-.344**	.121	-.256**	.446**	.527**	-.035	.527**	.575**	**.860****	-.303**	1.000

C1: Santolina triene; C2: α-Pinene; C3: Camphene; C4: β-Pinene; C5: Yomogi alcohol; C6: α-Terpienene; C7: p-Cymene; C8: Limonene; C9: Eucalyptol; C10: Artemisia ketone; C11: Sabinene hydrate; C12: Artemisia alcohol; C13: 3-Isopentenyl isovalerate; C14: Pinocarveol; C15: Camphor; C16: Pinocarvone; C17: Terpinen-4-ol; C18: α-Terpineol; C19: 3-Hexenyl isovalerate; C20: α-Longipinene; C21: α-Copaene; C22: Benzyl isovalerate; C23: β-Cubebene; C24: β-Caryophyllene; C25: β-Farnesene; C26: α-Humulene; C27: β-Chamigrene; C28: β-Selinene; C29: γ-Elemene; C30: Butylated- hydroxytoluene; C31: δ-Cadinene; C32: Caryophyllene oxide; C33: α-Muurolol; C34: Vulgarone B; C35: Lanceol

Bold letters- correlation coefficient > 0.6 or < -0.6.

* Statistically significant at the 5% significance level (p <.05)

** Statistically significant at the 1% significance level (p <.01)
